# Extracellular vesicles in atopic dermatitis: unraveling pathogenic mediators and engineering therapeutic vectors

**DOI:** 10.3389/fimmu.2026.1846799

**Published:** 2026-05-25

**Authors:** Wei-Zhen Tang, Chong-Yi Liao, Hong-Yu Xu, Wen-Ting Huang, Zhi-Xian Wu, Tong-Yu Chen, Tai-Hang Liu, Yong-Heng Wang

**Affiliations:** 1 Department of Laboratory Medicine, Chongqing Jiulongpo People’s Hospital, Chongqing, China; 2 Department of Dermatology, Xiangya Hospital, Central South University, Changsha, Hunan, China; 3 Department of Genetics and Cell Biology, School of Basic Medical Sciences, Chongqing Medical University, Chongqing, China; 4 Department of Dermatology, The Affiliated Shaoyifu Hospital of Zhejiang University, Hangzhou, Zhejiang, China

**Keywords:** atopic dermatitis, extracellular vesicles, mesenchymal stem cells, skin disease, Staphylococcus aureus

## Abstract

Atopic dermatitis (AD) is a complex chronic inflammatory skin disease whose pathogenesis involves a vicious cycle of epidermal barrier defects, immune dysregulation, and microbial imbalance. Despite advances in targeted biologics and small−molecule drugs, there remains an unmet clinical need for safe, effective treatments that can simultaneously intervene in multiple pathological processes. Extracellular vesicles (EVs), as key mediators of intercellular communication, play an increasingly prominent dual role in the pathophysiology and treatment of AD. This review systematically elaborates on this dialectical unity of EVs in AD. In terms of pathological mechanisms, EVs derived from pathogens such as *Staphylococcus aureus* and *Malassezia* spp., as well as from host mast cells, act as active “nanoscale pathological messengers” that deeply participate in disease initiation and progression by delivering virulence factors, disrupting the skin barrier, driving Th2/Th17 immune polarization, and sustaining chronic inflammation. Regarding therapeutic applications, therapeutic EVs derived from mesenchymal stem cells (e.g., adipose−, umbilical cord−derived), plants, probiotics, and marine organisms serve as efficient “cell−free therapeutic platforms, “ demonstrating great potential for intervening in AD through multipathway immunomodulation, active restoration of barrier function, and systemic regulation of the gut−skin axis. Moreover, strategies such as preconditioning parental cells (e.g., hypoxia, cytokine stimulation), genetic engineering, or constructing hybrid EVs can further enhance the therapeutic efficacy of EVs; combining them with advanced delivery systems (e.g., hydrogels, microneedles) can effectively overcome transdermal delivery bottlenecks. Although promising, the field still faces challenges including uneven source selection, lack of standardization in preparation and characterization, insufficient mechanistic elucidation, limitations of preclinical models, and inadequate pharmacokinetic and long−term safety data. Future research should focus on establishing standardized protocols, deepening mechanistic understanding, developing more accurate disease models, and exploring translational avenues to assess the potential for next−generation precision diagnostic and therapeutic strategies for AD based on EVs.

## 1 Introduction

Atopic Dermatitis (AD), commonly known as eczema, is the most prevalent chronic inflammatory skin disease worldwide. It is fundamentally a type I hypersensitivity syndrome, with a core characteristic being a genetic predisposition to produce high levels of immunoglobulin E (IgE)—a shared immunological basis for atopic conditions such as allergic asthma and hay fever (1). Clinically, AD is typified by intense pruritus, recurrent erythematous and exudative skin lesions, and a disease course marked by significant fluctuations and a chronic pattern (1).

From an epidemiological perspective, AD poses a substantial global disease burden. It is one of the most frequently diagnosed conditions in dermatological clinical practice (2). Data indicate that between 1990 and 2019, the number of prevalent cases of childhood AD globally increased by 5.7 million, with incident cases rising by a total of 700, 000 over the same period, highlighting its growing prevalence trend (3). Although AD often manifests in infancy and can persist into adulthood, it is noteworthy that approximately one-quarter of patients experience onset during adulthood. The estimated adult prevalence ranges from 2.1% to 4.9% and continues to rise (4, 5). Moderate-to-severe AD imposes significant direct and indirect economic burdens on patients, families, and society. Moreover, due to intractable pruritus, visible skin lesions, and resultant sleep disturbances, anxiety, and depression, it severely impairs patients’ quality of life and social functioning (6).

The pathophysiology of AD is highly complex, resulting from interactions among genetic, immune, and environmental factors (2). The currently established core pathogenesis can be summarized as a “triple hit”: dysfunction of the epidermal barrier, dysbiosis of the skin microbiome, and immune dysregulation dominated by T helper 2 (Th2) cells (7, 8). Specifically, inherited defects or functional impairment of key barrier proteins like filaggrin constitute the initial susceptibility basis for AD. The intense, often unbearable pruritus leads to repetitive scratching, causing further mechanical damage and collectively compromising the skin’s physical defense (8). This barrier dysfunction not only increases transepidermal water loss but also elevates skin pH, thereby disrupting the skin microbiome balance, affecting protease activity, and promoting the release of inflammatory and pruritogenic mediators (9). The compromised epidermis facilitates the penetration of environmental allergens and antigens from resident skin microbes. Notably, the over-colonization and invasion of *Staphylococcus aureus* (*S. aureus*) and *Malassezia sympodialis* in AD lesions play key pathogenic roles (10, 11). These antigens interact with immune cells and stimulate keratinocytes to release alarmins such as TSLP and IL-33, initiating and amplifying aberrant immune responses (1, 10). Dendritic cells (DCs) capture and present antigens, driving naïve T cell differentiation toward a Th2 phenotype. Activated Th2 cells secrete core cytokines like IL-4, IL-5, and IL-13, which induce IgE class switching in B cells, leading to the production of large amounts of antigen-specific IgE (8). IgE binds to the high-affinity receptor FcϵRI on mast cells and basophils, triggering their degranulation and the release of mediators such as histamine and leukotrienes, thereby inducing acute inflammation and intense pruritus. Furthermore, macrophages recruited to lesional skin tend to polarize into an M2 phenotype induced by IL-4 (which promotes Th2 responses), rather than the classically activated M1 phenotype, further consolidating the Th2-skewed inflammatory microenvironment in AD (12, 13).

Confronting this complex vicious cycle formed by “barrier defect–immune dysregulation–microbial disturbance, “ a definitive cure for AD remains an unmet major clinical need (14). Traditional stepwise therapeutic strategies focus on long-term disease control, relapse prevention, and symptom relief. Topical corticosteroids are the first-line treatment for controlling acute inflammation, while topical emollients aim to restore barrier function (15, 16). For moderate-to-severe patients, systemic immunosuppressants (e.g., cyclosporine) or topical calcineurin inhibitors (e.g., tacrolimus) can be used to inhibit T cell activation, but their use is often limited due to potential systemic or local side effects (16–18). In recent years, based on a deeper understanding of the molecular pathways in AD pathogenesis, targeted biologics (e.g., the anti-IL-4/IL-13 receptor monoclonal antibody Dupilumab) and small-molecule JAK-STAT inhibitors have shown great promise and have been approved for clinical use (19, 20). However, challenges remain, including incomplete coverage of all patient subtypes, need for further observation of potential long-term safety, and high treatment costs (18).

Against this backdrop, advances in tissue engineering and regenerative medicine offer new perspectives for AD treatment. Cell-based therapies, such as those involving Mesenchymal Stem Cells (MSCs), have garnered significant interest due to their potent immunomodulatory and tissue repair capabilities (21, 22). However, live cell transplantation faces inherent challenges, including low homing efficiency, uncertain survival, potential tumorigenic risk, complex manufacturing processes, and stringent regulatory requirements (23). Research suggests that the primary therapeutic effects of stem cells are attributed to their paracrine actions (24). Since key discoveries in the early 2000s confirmed that Extracellular Vesicles (EVs)—lipid bilayer-enclosed nanoparticles actively released by cells—can transfer functional proteins, lipids, and nucleic acids (e.g., mRNA, miRNA) between cells, thereby serving as crucial mediators of intercellular communication, interest in EV-based therapies has surged (25–27). Compared to live cells, EVs offer unique advantages: they are unable to replicate, exhibit high biocompatibility, are easier to manufacture at scale and store in a standardized manner, and can cross physiological barriers. This makes EVs a highly promising ideal platform for “off-the-shelf” cell-free therapies, capable of replicating the beneficial paracrine effects of cells like MSCs while circumventing many risks associated with whole-cell therapies (28–30).

Notably, within the pathological context of AD, EVs play a dual and opposing role. On one hand, EVs derived from pathogens (e.g., S. aureus, Malassezia) and host immune cells (e.g., mast cells) act as active “nanoscale pathological messengers, “ directly participating in delivering virulence factors, disrupting the skin barrier, driving Th2 immune polarization, and sustaining chronic inflammation. This reveals a novel and critical dimension in the pathogenesis of AD. On the other hand, therapeutic EVs derived from mesenchymal stem cells (e.g., adipose-, umbilical cord-derived), plants, probiotics, and even marine organisms serve as efficient “therapeutic vectors, “ demonstrating great potential in preclinical models for intervening in AD through mechanisms such as multi-pathway immunomodulation, active restoration of barrier function, and systemic regulation of the gut-skin axis.

Therefore, this review aims to systematically elucidate this duality of EVs in the field of AD. First, it will analyze how EVs, as emerging pathological mediators, deeply participate in and exacerbate the initiation and progression of AD. Second, it will comprehensively review the mechanisms of action, therapeutic prospects, and preclinical evidence supporting the potential for future translation of therapeutic EVs from various sources and their engineered strategies. Finally, based on the core limitations of current research, it will outline key future directions. By integrating the two main threads of “pathological mechanisms” and “therapeutic applications, “ this review hopes to provide a novel perspective for understanding the complex pathological network of AD and lay a solid theoretical foundation for developing next-generation, EV-based precision diagnostic and therapeutic strategies for AD.

## 2 Definition and classification of EVs

EVs are lipid bilayer-enclosed particles actively released by cells into the extracellular space and incapable of self-replication. EVs are widely involved in intercellular communication, tissue homeostasis, and the regulation of pathological processes (31, 32). Given the remarkable heterogeneity of EVs with respect to their biogenesis mechanisms, particle size, density, cellular origin, molecular composition, and functional properties, precise terminology is critical for the accurate interpretation of findings across studies. The International Society for Extracellular Vesicles (ISEV) recommends that, in the absence of definitive evidence regarding biogenesis, the umbrella term “extracellular vesicles” should be preferentially adopted (33–35). By contrast, vesicle-like structures obtained through artificial processes—such as tissue disruption, cell extrusion, or sonication—may recapitulate certain morphological or functional features of EVs, but should not be equated directly with naturally occurring EVs (36, 37).

Based on the classical classification, naturally occurring EVs are broadly categorized into three major subtypes: exosomes, microvesicles, and apoptotic bodies (38, 39). The most commonly studied are exosomes, typically ranging from 30 to 200 nanometers in diameter. They originate from a complex endocytic pathway: inward budding of the plasma membrane forms early endosomes, which mature into multivesicular bodies enriched with intraluminal vesicles. Ultimately, these multivesicular bodies fuse with the plasma membrane, releasing their intraluminal vesicles as exosomes into the extracellular space (40). This process is tightly regulated by mechanisms involving the endosomal sorting complex, and their membranes are characteristically enriched with tetraspanins CD9, CD63, CD81, and TSG101, while excluding cytoplasmic proteins, annexins, miRNA-processing enzymes, double-stranded RNA and other contaminants commonly misclassified as exosomal components. All remaining poorly characterized vesicle populations are uniformly referred to as EVs (41). The second class comprises microvesicles, also known as ectosomes, with a diameter of approximately 100–1000 nanometers, formed through direct outward budding of the plasma membrane (42, 43). Unlike the endosomal origin of exosomes, microvesicle formation depends on plasma membrane remodeling, cytoskeletal rearrangement, calcium signaling alterations, and changes in membrane phospholipid asymmetry. The third class is apoptotic bodies, which can reach 100–2000 nanometers in diameter and are generated during the late stages of apoptosis, encapsulating intact cytoplasmic components (44). Additionally, microbially derived EVs constitute a distinct category of naturally occurring EVs: Gram-negative bacteria produce outer membrane vesicles via budding from their outer membrane, whereas Gram-positive bacteria generate vesicles originating from their cytoplasmic membrane (45).

Despite this classical classification framework, the rigorous discrimination of distinct EV subtypes remains technically challenging. Considerable overlap exists among EV subtypes with respect to particle size, density, membrane composition, and molecular markers. Moreover, current isolation and purification techniques are generally insufficient to fully preserve or resolve their true biogenetic origins (33–35). Consequently, the use of EVs as an umbrella term remains the more prudent and standardized approach in most functional studies, a convention followed in this review a convention followed in this review (36, 46).

Beyond naturally secreted EVs, non-canonical or artificially prepared EV-like vesicles have been increasingly employed in recent years for mechanistic disease research, drug delivery, and regenerative medicine. Depending on their origin and preparation method, these entities are classified as nanovesicles, EV-mimetics, or engineered hybrids. Nanovesicles (NVs) generally refer to nanoscale lipid bilayer structures derived from plant tissues, cell membranes, or other biological source materials through processes such as homogenization, mechanical disruption, extrusion, or ultracentrifugation (47–49). For example, plant-derived exosome-like nanovesicles (PELNs) can be isolated from plant tissues such as Portulaca oleracea (purslane) and grapefruit, typically falling within a size range of approximately 30–300 nm, and exhibiting characteristics—including morphology, stability, and select biological functions—comparable to those of EVs derived from mammalian cells (49, 50).

EV-mimetics (EVMs) represent another important class of EV-like systems, typically prepared through sequential cell extrusion, sonication, microfluidic processing, or other top-down engineering strategies (51, 52). Compared with naturally occurring EVs, EVMs offer notable advantages in terms of higher yield and greater scalability of the production process. Furthermore, EVMs can retain, to a considerable extent, the membrane proteins, lipid composition, and biological activity of the source cells, thereby providing an alternative strategy to address the challenges of high heterogeneity, low yield, and limited clinical translatability that constrain the application of natural EVs (37).

Engineered hybrids generally refer to composite delivery systems assembled through deliberate engineering strategies, in which naturally occurring EVs, nanovesicles, cell membrane-derived vesicles, liposomes, therapeutic molecules, targeting ligands, or other synthetic materials are fused, modified, or assembled into integrated constructs (53, 54). In this context, “engineered” emphasizes the deliberate interventions involved—whether genetic, chemical, physical, microfluidic, or membrane-based—while “hybrid” underscores the fact that these systems are composed of two or more biological or synthetic components. Unlike unmodified natural EVs, engineered hybrids typically possess artificially designed membrane architectures, targeting capabilities, drug-loading properties, or immunomodulatory functions, and their biological effects frequently arise from the synergistic contributions of multiple components rather than from any single natural EV constituent (55, 56).

## 3 Biological characteristics of EVs

Based on the classification framework outlined above, the biological significance of EVs is primarily determined by the collective contributions of their molecular cargo, lipid bilayer architecture, extracellular stability, *in vivo* biodistribution, and capacity to interact with recipient cells (31, 32). Far from being mere “garbage bags” for cellular waste disposal as initially perceived, EVs are now recognized as active carriers playing crucial messenger roles in normal physiological homeostasis and numerous pathological processes (32, 57). By encapsulating and delivering bioactive “cargo” such as proteins, lipids, and nucleic acids to neighboring or distant target cells, EVs may modulate the functions of the latter. This characteristic has garnered significant attention for EVs in the fields of regenerative medicine, disease mechanism research, and therapeutic development (58–60).

The functional basis of EVs lies in their complex and dynamically changing molecular composition. This composition acts as a “molecular fingerprint” of their parental cells, profoundly reflecting the cell type, state, and microenvironment (61). Their lumens are rich in diverse bioactive molecules: in terms of proteins, this includes growth factors, cytokines, and enzymes performing specific functions. Their lipid composition is characterized by a high proportion of ceramides, sphingomyelins, cholesterol, and phosphatidylserine, which not only confer structural stability to the vesicle membrane but are also involved in cellular recognition processes (62–64). Particularly crucial is their nucleic acid cargo, encompassing DNA, mRNA, and non-coding RNAs (such as miRNAs, lncRNAs, and circRNAs), which have been implicated in the modulation of gene expression networks at the post-transcriptional level (65–67). The delivery of these RNA molecules via EVs has been associated with alterations in target cell proliferation, differentiation, metabolism, apoptosis, and microenvironment remodeling, suggesting a potential contribution to EVs’ long-range regulatory functions (68). This dynamic composition holds significant biological implications. For instance, the specific elevation of miR-199a-3p in serum EVs from patients with psoriasis correlates positively with disease severity, making it a potential diagnostic biomarker (69). Furthermore, EVs derived from three-dimensional culture systems may contain more abundant pro-repair factors compared to those from two-dimensional cultures, thereby exhibiting stronger therapeutic activity (70, 71).

From biogenesis to functional realization, EVs undergo a precisely regulated life cycle. Their biogenesis begins with early sorting endosomes formed via endocytosis. Through material exchange and maturation, the endosomal membrane invaginates to form intraluminal vesicles, which are then encapsulated within multivesicular bodies (72). Subsequently, multivesicular bodies are transported to the plasma membrane with the aid of the cytoskeleton and fuse with it, releasing EVs via exocytosis (73). Once released extracellularly, EVs are transported via bodily fluids and are taken up by target cells through multiple mechanisms: these include direct signal activation dependent on specific ligand-receptor interactions, complete fusion of the EV membrane with the target cell membrane, and internalization primarily via various endocytic pathways such as clathrin-dependent, caveolin-dependent, or macropinocytosis pathways (74, 75). These pathways often work synergistically to ensure efficient cargo delivery (58). Upon entering the cell, EVs or their contents may be released into the cytoplasm, potentially influencing the target cell’s phenotype and function. Ultimately, this process has been implicated in various physiological and pathological processes, including tissue repair, immune regulation, and metabolic homeostasis.

In summary, EVs represent a well-defined class of nanoscale communication systems with specific biogenesis pathways, carrying heterogeneous molecular cargo and following complex uptake and action mechanisms. As a universal “language” for intercellular information exchange, their highly plastic composition and function form the basis for their deep involvement in complex disease processes like AD. A thorough understanding of these core biological characteristics is fundamental to subsequently dissecting the specific roles of EVs in the pathological mechanisms of AD and evaluating their potential for diagnostic and therapeutic applications.

## 4 The pathophysiological mechanisms of EVs in the onset and progression of AD

**Figure 1:**
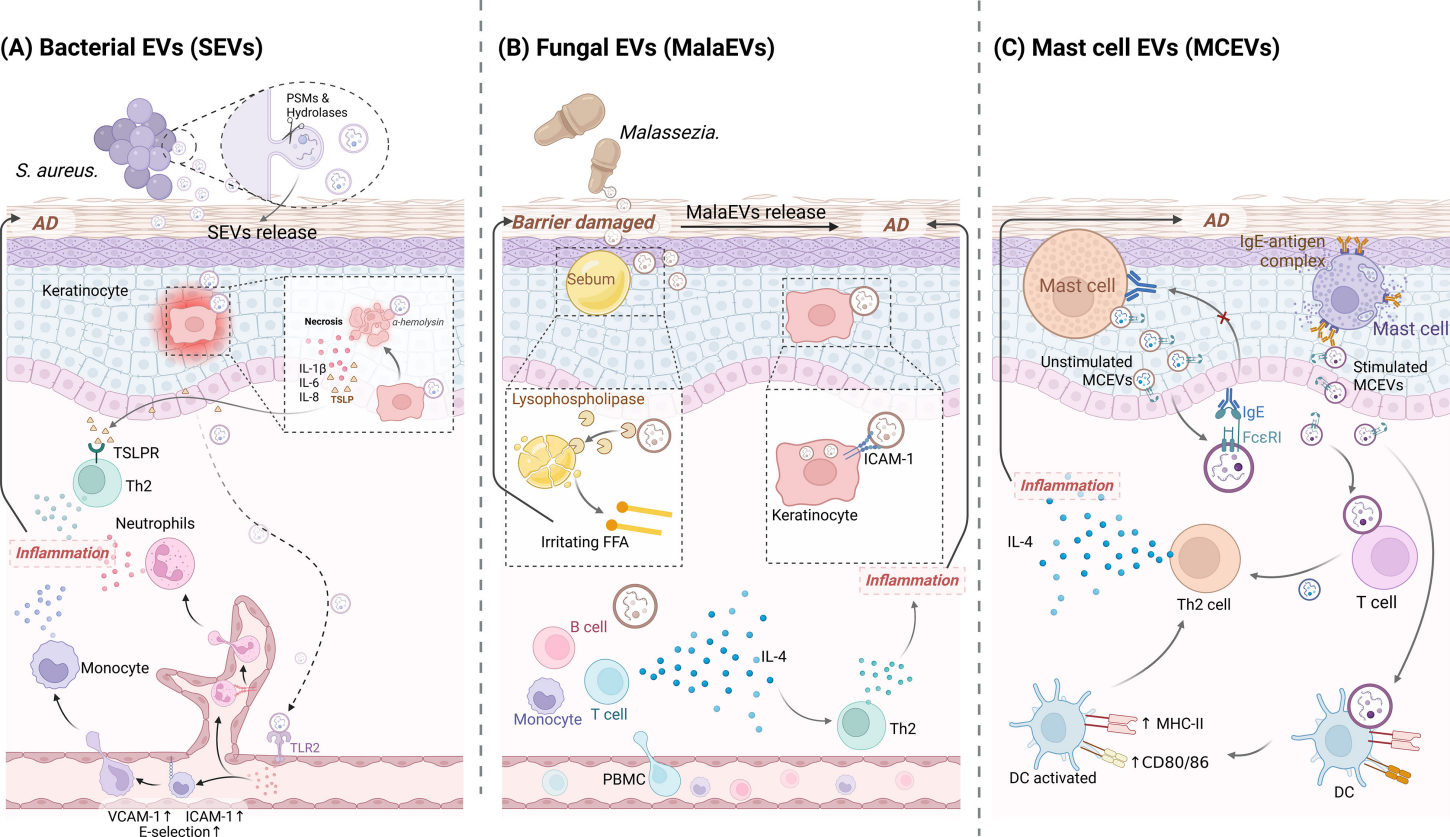
Pathogenic mechanisms mediated by SEVs, MalaEVs, and MCEVs in AD **(A)** SEVs **(B)** MalaEVs **(C)** MCEVs. **(A)** Staphylococcus aureus-derived EVs (SEVs). Staphylococcus aureus releases SEVs through the synergistic action of PSMs and hydrolytic enzymes. Alpha-toxin directly induces keratinocyte necrosis, thereby disrupting the skin barrier. Concurrently, SEVs stimulate keratinocytes to release TSLP, IL-1β, IL-6, and IL-8, which activate Th2 immune responses. SEVs engage TLR2 on dermal microvascular endothelial cells, leading to upregulation of VCAM-1, ICAM-1, and E-selectin, which in turn promotes the recruitment of neutrophils and monocytes and drives local inflammatory responses. **(B)** Malassezia-derived EVs (MalaEVs). Following disruption of the skin barrier, Malassezia releases MalaEVs. Vesicle-associated phospholipases hydrolyze sebum lipids to generate irritant free fatty acids, which compromise the lipid architecture of the stratum corneum. Upon internalization by keratinocytes, MalaEVs induce ICAM-1 expression. Simultaneously, MalaEVs stimulate PBMCs to produce IL-4, thereby promoting Th2 cell differentiation and converting commensal colonization into sustained Th2-type allergic inflammation. **(C)** Mast cell-derived EVs (MCEVs). Mast cells constitutively secrete unstimulated MCEVs under resting conditions. Upon activation via crosslinking of the FcϵRI by IgE-antigen complexes, mast cells release stimulated MCEVs. Stimulated MCEVs promote IL-4 production by Th2 cells and induce DC maturation—characterized by upregulation of MHC class II and CD80/86—thereby enhancing antigen-presenting capacity. This establishes a positive feedback loop that amplifies Th2-type inflammatory responses in AD.

Within the increasingly clarified pathophysiological landscape of AD, a novel axis is garnering attention: a complex communication network formed by EVs derived from diverse cellular sources. This network transcends the traditional mode of action of soluble factors, and may contribute to disease initiation and progression. Specifically, EVs released by dysbiotic skin microbes (such as *Staphylococcus aureus* and *Malassezia* spp.) act as actively deployed “nanoscale virulence vehicles, “ capable of directly traversing the compromised skin barrier to deliver concentrated effector molecules, thereby profoundly reshaping the local immune microenvironment. Concurrently, activated immune cells within the host (e.g., mast cells) also secrete functionally specialized EVs. These serve as dynamic immunomodulatory messengers, playing complex and variable roles in either promoting inflammation or attempting to restore homeostasis. These two categories of EVs—originating from pathogens and the host itself—interweave to form a multi-dimensional, multi-target pathological messenger system, influencing the disease characteristics of AD across multiple levels, from barrier disruption and immune polarization to inflammatory cascades (**Figure 1**).

### 4.1 Staphylococcus aureus-derived EVs

SEVs have been implicated as potential contributors to the pathological process of AD. Their very release challenges traditional understanding, as the thick cell wall of Gram-positive bacteria was once thought to hinder EV release. However, successful secretion of SEVs relies on phenol-soluble modulins (PSMs) increasing membrane fluidity and the activity of cell wall hydrolases (such as N-acetylmuramyl-L-alanine amidase) carried by the vesicles themselves; these mechanisms collectively facilitate their passage through the cell wall barrier (76, 77). Within the unique microenvironment of AD skin (e.g., elevated pH, reduced antimicrobial peptides and filaggrin), SEVs act as an extension of bacterial virulence, playing multiple destructive roles. Proteomic analyses confirm that SEVs are a concentrated “repository of virulence factors, “ containing superantigens, α-hemolysin, coagulase, and immunomodulatory proteins like Staphylococcal Protein A (SpA) (76). Encapsulated within the EVs’ lipid bilayer, these factors gain significant stability against degradation by host proteases and achieve effective targeted delivery into deeper epidermal layers. For instance, SpA can be detected throughout the full thickness of the epidermis in AD lesions, a phenomenon absent in healthy skin, providing direct evidence of the superior skin-penetrating capability of SEVs (78). Their pathogenic mechanisms are primarily manifested at three levels: First, direct assault on the physical and cellular skin barrier. The α-hemolysin carried by SEVs exhibits potent cytotoxicity against keratinocytes, inducing rapid cell necrosis. This leads to the collapse of epidermal barrier function and triggers compensatory hyperplasia (lichenification), a typical pathological feature of AD (79). Second, potential modulation of local immune homeostasis. SEVs have been shown to induce activation of both epidermal keratinocytes and dermal fibroblasts *in vitro*, leading to increased release of key mediators such as thymic stromal lymphopoietin (TSLP), IL-1β, IL-6, IL-8, and eotaxin (78, 80). TSLP is the “master switch” for initiating Th2-type immune responses, while IL-6 inhibits the normal terminal differentiation of keratinocytes, further weakening barrier function (81). Simultaneously, SEVs activate dermal microvascular endothelial cells via the TLR2/4-NF-κB signaling pathway, upregulating the expression of adhesion molecules like E-selectin, VCAM-1, and ICAM-1. This potently recruits immune cells such as neutrophils and monocytes to infiltrate the dermis, exacerbating inflammation (82, 83). Third, association with systemic immune polarization. This multi-cellular, multi-factor synergistic action has been associated with the typical Th2/Th17 mixed inflammatory response seen in AD, accompanied by a significant elevation in both total serum IgE and levels of IgE specific to SEVs (80, 84, 85). Furthermore, SEVs consolidate their pathogenic status indirectly by altering skin surface properties through biofilm precursor formation, aiding bacterial colonization and resisting competition from other microbes, while also protecting bacteria from clearance by host phagocytes (86, 87). More notably, SEVs can carry active molecules like β-lactamase, horizontally disseminating antibiotic resistance among bacterial populations, which poses an additional challenge for eradicating *S. aureus* in AD treatment (88). From a translational medicine perspective, the elevation of SEV-specific IgE in the serum of AD patients, along with the diagnostic potential demonstrated by serum microbiome-derived EV metagenomic analysis, elevates SEVs from mere local inflammatory mediators to the status of systemic immune disturbance biomarkers and potential therapeutic targets (85).

### 4.2 Malassezia-derived EVs

MalaEVs elucidates the unique mechanism of fungus-host interaction in AD pathology. As a resident, lipophilic yeast on the skin, *Malassezia sympodialis* over-proliferates in the lesions of AD patients. The EVs it releases serve as a key medium for breaking immune tolerance and inducing Th2-type allergic reactions. The release of MalaEVs also requires overcoming the thick fungal cell wall. Hydrolytic enzymes enriched within their lumen are thought to potentially facilitate release by locally digesting cell wall components (89, 90). Proteomic analysis of MalaEVs reveals its highly complex composition, with over 2400 identified proteins. Among these, hydrolases (such as lysophospholipase) and known allergens (e.g., Mala s1, Mala s5-13) are significantly enriched (89, 91). These components confer upon MalaEVs a dual pathogenic capacity. On one hand, they may compromise the skin’s chemical barrier. The high concentration of lysophospholipase in the vesicles has the capacity to catalyze the decomposition of sebum. The resulting irritating free fatty acids disrupt the stratum corneum lipid structure, increase transepidermal water loss, and directly compromise skin barrier integrity (92, 93). On the other hand, they have been associated with the promotion of a Th2-skewed immune response. Studies confirm that MalaEVs can be effectively internalized by keratinocytes, stimulating them to highly express intercellular adhesion molecule-1 (ICAM-1), thereby enhancing the adhesion and retention of immune cells at inflammatory sites (89). More critically, MalaEVs can potently and specifically induce the production of IL-4 from peripheral blood mononuclear cells (PBMCs) of atopic individuals. IL-4 is the core cytokine driving IgE production by B cells and promoting the differentiation of naïve T cells toward a Th2 phenotype (94). Concurrently, MalaEVs can also elicit a general TNF-α response, exacerbating local inflammation. These effects indicate that EVs from *Malassezia*, which might otherwise exist in a commensal state, become a critical bridge converting mere microbial colonization into a persistent, IgE-mediated, Th2-type allergic inflammation.

### 4.3 Mast cell-derived EVs

MCEVs possess unique biological characteristics. They exist as cup-shaped structures approximately 60–100 nm in diameter. Interestingly, these vesicles have been found pre-stored within cytoplasmic granules, providing a structural basis for their rapid and abundant release upon antigen exposure, making them part of the mast cell’s immediate response mechanism (95). Depending on the cell’s activation state, mast cells secrete two functionally distinct EV subpopulations: “unstimulated MCEVs” constitutively secreted under resting conditions, and “stimulated MCEVs” secreted following activation via antigen-IgE-FcϵRI cross-linking (96). This distinction is not merely quantitative but qualitative. Proteomic and lipidomic analyses reveal that stimulated MCEVs exhibit high surface expression of the lysosome-associated membrane protein CD63 and are enriched with granule-derived proteases (e.g., CPA3, mMCP4/5/6) and phosphatidylinositol. This suggests they may originate directly from the degranulation process and serve as an efficient delivery system for pre-formed mediators stored within granules (96). Notably, regardless of stimulation, MCEVs carry antigen-presentation-associated molecules, including MHC class II molecules, co-stimulatory molecules (CD86, CD40), and adhesion molecules (LFA-1/ICAM-1), endowing them with potential antigen-presenting and immune cell-activating capabilities (95).

MCEVs exhibit functional bidirectionality and complexity in AD, with their effects likely contingent on the microenvironment, disease stage, and target cell type. On one hand, multiple lines of evidence support a pro-inflammatory and Th2-promoting role for MCEVs, aligning with the classic pathology of AD. *In vitro* studies demonstrate that MCEVs can promote T lymphocyte proliferation and are associated with their differentiation toward a Th2 phenotype, along with increased production of Th2-type cytokines, suggesting a potential role in the characteristic Th2 inflammation in AD (97). Furthermore, MCEVs can significantly promote the production of IL-5 by type 2 innate lymphoid cells (ILC2s), further amplifying type 2 immune responses (98). Concurrently, MCEVs have been shown to induce maturation of dendritic cells (DCs), as evidenced by upregulation of surface MHC class II and co-stimulatory molecules (CD80, CD86, CD40) on DCs, enhancing their antigen-presenting capacity. Moreover, DCs that have internalized MCEVs can more efficiently present antigens carried by the vesicles to T cells, eliciting stronger T cell activation—a property not shared by EVs from B cells or macrophages, highlighting the unique role of MCEVs in initiating adaptive immunity (99).

On the other hand, research has also unveiled potentially immunomodulatory and even protective aspects of MCEVs. A key finding is that MCEVs express the FcϵRI receptor on their surface, enabling them to directly bind and neutralize free IgE, thereby reducing serum levels of free IgE. When IgE is pre-bound by MCEVs, its ability to trigger mast cell degranulation is significantly inhibited. This suggests MCEVs may function as an “IgE sponge, “ potentially contributing to the feedback regulation of allergic reaction intensity and attenuating over-activation (100). Even more intriguingly, an *in vivo* study yielded results seemingly contradictory to *in vitro* experiments: injecting mice with MCEVs was associated with a Th1-type response (e.g., IFN-γ production) rather than a Th2 response (95). In the context of AD, a moderate Th1 response could exert an inhibitory effect on an overactive Th2 response. This discrepancy between *in vitro* and *in vivo* findings may stem from the complex cellular networks and regulatory mechanisms *in vivo*, indicating that the ultimate immunologic effect of MCEVs is highly dependent on the physiological context in which they operate.

## 5 EVs as interconnected nodes in AD pathogenesis and therapy

As demonstrated above, pathogen-derived EVs (SEVs, MalaEVs) and host immune cell-derived EVs (MCEVs) constitute an interconnected signaling network that drives barrier disruption, Th2/Th17 polarization, and inflammatory amplification in AD. Yet the mechanisms by which therapeutic EVs counteract these pathological processes remain incompletely defined.

Recent studies indicate that EVs function as central mediators of intercellular communication, coordinating immune responses, tissue remodeling, and disease progression through transfer of bioactive cargo (101, 102). In inflammatory disorders, EVs modulate NF-κB and JAK/STAT signaling to sustain chronic inflammation and shape microenvironmental dynamics (103–105). This raises the question of whether therapeutic EVs disrupt AD pathogenesis by interfering with pathogenic EV-mediated circuits rather than acting as isolated anti-inflammatory agents.

Emerging evidence supports multi-level intervention by therapeutic EVs in AD. First, they restore epithelial barrier integrity through delivery of barrier lipid precursors and upregulation of structural proteins, thereby blocking DAMP release and subsequent inflammatory cascade initiation (106–108). Second, therapeutic EVs suppress NF-κB, MAPK, and JAK/STAT signaling while reducing Th2 cytokine production and promoting Treg differentiation and M2 macrophage polarization, thus counteracting pathogenic EV-driven immune polarization (109, 110). Third, therapeutic EVs deliver anti-inflammatory miRNAs that target IRAK1, TRAF6, and NLRP3, enabling post-transcriptional reprogramming of recipient cells (111–115). These findings suggest that therapeutic EVs exert disease-modifying effects by reconfiguring pathogenic signaling networks rather than simply antagonizing individual inflammatory mediators.

The subsequent sections examine therapeutic EVs derived from distinct cellular and biological sources, focusing on their capacity to disrupt specific pathological circuits in AD.

## 6 Current status of research and therapy for AD utilizing EVs

The therapeutic application of EVs in AD must be considered in the context of the disease’s pronounced clinical and immunological heterogeneity. AD has evolved conceptually from a simple phenotypic classification toward a mechanistically grounded “endotype”-based framework (15, 116). Traditionally, AD has been divided into extrinsic (characterized by elevated IgE and Th2 dominance) and intrinsic (characterized by normal IgE levels with Th1/Th17 involvement) subtypes (117, 118); however, the disease in practice engages multiple immune axes, with considerable variation across individual patients, disease stages, and ethnic populations (116, 119). Beyond Th2 dominance, multiple immune axes—including Th1, Th17, and Th22—contribute variably across patients, disease stages, and populations. For instance, Th2/Th22 polarization is a prominent feature of acute atopic dermatitis, and pediatirc AD is characterized by a high activation of Th2 and Th17 cells (118, 119).

Therapeutic EV strategies should be tailored to the specific endotype in question. For Th2-high endotypes, MSC-derived EVs represent a particularly appropriate approach, given their capacity to suppress type 2 cytokines and promote barrier restoration (120, 121). Intrinsic AD, by contrast, may be more amenable to engineered EVs designed to modulate broader inflammatory pathways (122, 123), while dysbiosis-associated AD may benefit from probiotic- or plant-derived EVs (124, 125). Although the supporting evidence remains largely at the preclinical stage, an endotype-guided framework holds considerable value for directing the clinical translation of EV-based therapies (120, 126). The following sections summarize EV-based therapeutic strategies organized by biological origin and discuss their respective applicability across AD endotypes.

### 6.1 EVs derived from mesenchymal stem cells

**Figure 2:**
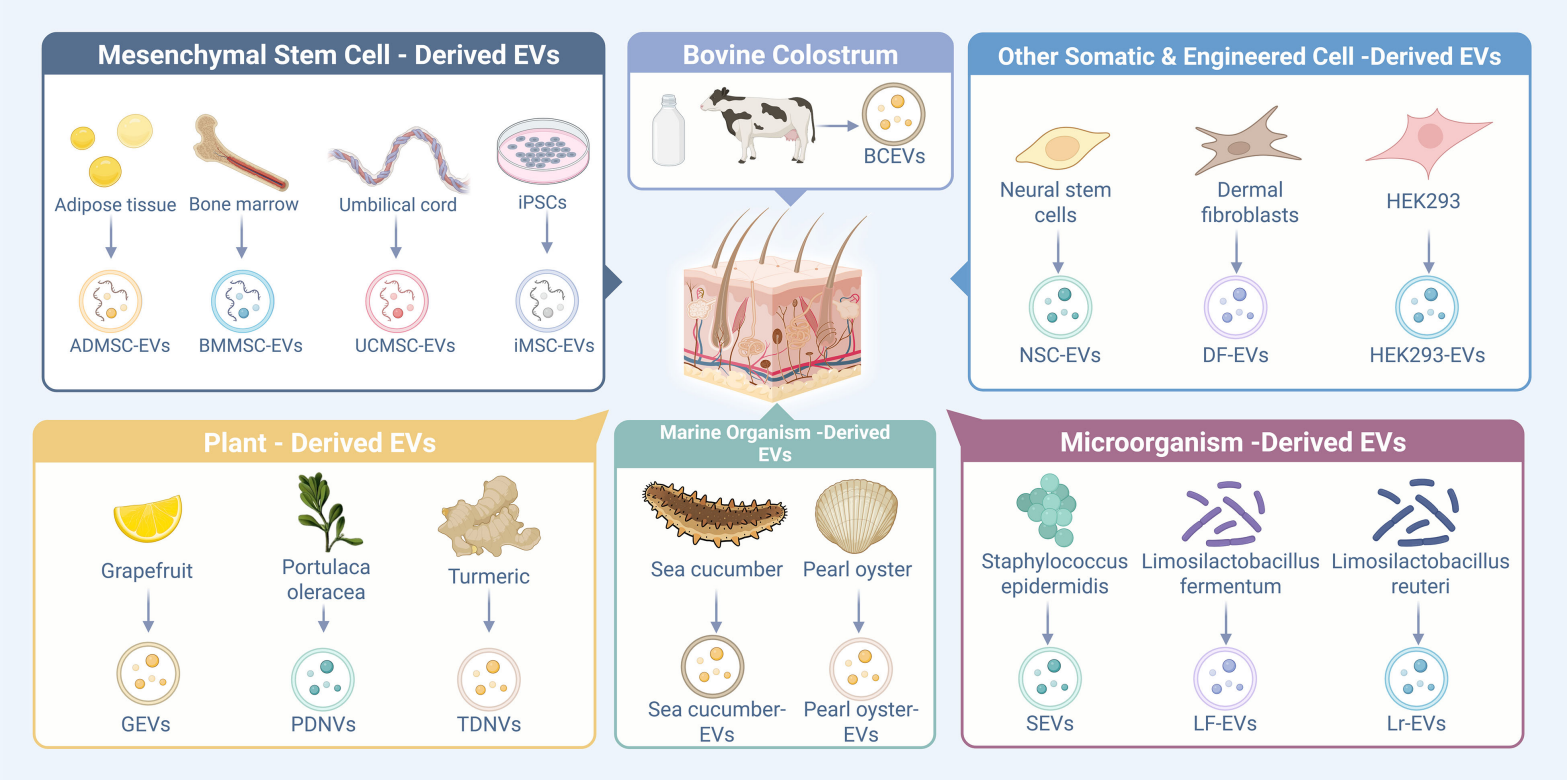
Current status of research and therapy for AD utilizing EVs.

EVs derived from MSCs represent the most extensively studied and diverse cell-free strategy for AD therapy. Their core advantage lies in the fact that MSC-EVs from different tissue sources (e.g., adipose, umbilical cord, bone marrow) not only inherit the potent multi-target immunomodulatory and tissue repair capabilities of their parental cells but also offer a robust platform for transitioning from in-depth mechanistic research to clinically translatable therapeutic products. This is due to their ease of acquisition, potential for large-scale production, and high degree of engineerability (e.g., customizing their “cargo” through preconditioning or genetic modification) (127, 128) (**Table 1**; **Figure 2**). Studies have confirmed that MSC-derived EVs may be particularly relevant to Th2-high, extrinsic, and barrier-defective AD, while specific MSC-EV subsets may also address mixed inflammatory endotypes involving Th1/Th17 activation (120, 121).

**Table 1:** Summary of study characteristics and outcomes for MSCs derived EVs in AD.

Author	Year	Country	EVs source	Isolation methods	Modification	Cargoes	Outcome (*in vitro* or ex vivo)	*In vivo* (delivery route)	Outcome (*in vivo*)
Shin et al. (129)	2020	South Korea	ASCs	Tangential flow filtration with ExoSCRT technology	None	Proteins (Alix, TSG101, CD9, CD81); enriched in ceramides, dihydroceramides, sphingolipids, non−esterified fatty acids; contains lipid-metabolizing enzymes	Enhances lamellar body formation and ceramide synthesis; suppresses inflammatory cytokines (IL−4, IL−5, IL−13, TNF−α, IFN−γ, IL−17, TSLP); promotes keratinocyte differentiation and barrier−related gene expression	Subcutaneous injection	Reduces epidermal hyperplasia and erythema; decreases TEWL; increases stratum corneum hydration; lowers inflammatory cytokines and IgE; elevates ceramide and dihydroceramide levels; restores lamellar bilayer structure at SG−SC interface
Park et al. (130)	2022	South Korea	ASCs	Tangential flow filtration with ExoSCRT technology	None	Contains exosomal markers (Alix, TSG101, CD9, CD81); surface markers analyzed by MACSPlex kit; enriched in lipid metabolism-related components	In murine models: suppresses Th2 cytokines (IL-4, IL-31, TNF-α, etc.); promotes keratinocyte barrier repair; enhances ceramide synthesis	Topical application assisted by transdermal electroporation	Significant improvement in dupilumab-related facial redness in two AD patients; well tolerated with no adverse effects; reduced erythema severity and extent
Han et al. (131)	2023	South Korea	ASCs	Tangential flow filtration with ExoSCRT technology	None	Contains immunomodulatory and regenerative factors (specific cargoes not detailed)	Not performed	Topical application with sonophoresis (5 weekly treatments)	Improved investigator global assessment and erythema scores; reduced IL-1α and TSLP; increased filaggrin and VEGF in stratum corneum; high patient satisfaction.
Shi et al. (111)	2023	China	ASCs	ExoQuick-TC kit	miR-147a overexpression	High expression of miR-147a; may contain other immunomodulatory factors	Suppresses TNF-α/IFN-γ-induced HaCaT inflammation/apoptosis; inhibits HUVEC angiogenesis; downregulates VEGFA and MEF2A-TSLP axis	Topical application (mouse ear)	Reduces ear swelling; inhibits skin inflammation and angiogenesis; downregulates VEGFA and TSLP expression
Roh et al. (132)	2024	South Korea	ASCs	Tangential flow filtration with ExoSCRT technology	None	Contains various regenerative and immunomodulatory factors.	Reduces pro-inflammatory cytokines (IL-6, IL-1β, IL-1α); increases anti-inflammatory IL-10 and skin barrier proteins (filaggrin, loricrin) in a triple-cell AD-like model.	Not performed	Not applicable
Shin et al. (133)	2025	South Korea	ASCs	Tangential flow filtration with ExoSCRT technology	None	Enriched in free fatty acids, ceramides, sphingomyelin; contains acidic sphingomyelinase, SPHK1, and other lipid-metabolizing enzymes.	Promotes keratinocyte uptake; suppresses Th2 cytokines (IL-1β, IL-4, IL-31); restores S1P levels; enhances keratinocyte differentiation.	Topical application	Reduces skin inflammation; improves epidermal barrier function; increases ceramide and S1P levels in AD mouse model
Wan et al. (134)	2025	South Korea	ASCs	Not specified in detail	None	Bioactive molecules (growth factors, cytokines, lipids, microRNAs)	Not performed	Topical application (twice daily for 6 weeks)	Improved vIGA−AD score (85% patients with ≥1−point reduction); increased skin hydration (+58%); reduced TEWL (−42%); decreased pruritus VAS score (−70%) in patients with facial AD
Park et al. (135)	2025	South Korea	WJ-MSCs	3D microwell−based platform + tangential flow filtration	None	Contains miR−146a and other immunomodulatory molecules	In TNF−α−induced HaCaT cells: suppresses AKT/NF−κB and MAPK pathways via miR−146a/IRAK1 axis; reduces Th1 cytokines (TNF−α, IFN−γ)	Single subcutaneous injection	Improves dermatitis score and epidermal thickness; reduces Th2, Th1, Th17, and Th22 cytokines; suppresses TSLP expression; restores filaggrin/occludin; comparable or superior to dupilumab with broader immunomodulation.
Park et al. (136)	2025	South Korea	WJ-MSCs	Tangential flow filtration (from 3D spheroid culture)	None	Surface markers (CD9, CD63, CD81); contains various bioactive molecules (proteins, RNAs, etc.)	Not performed	Single subcutaneous injection	Alleviates AD symptoms (erythema, edema, pruritus); reduces serum IgE and Th2 cytokines (IL−4, IL−13); modulates splenic CD4+/CD8+ ratio; restores skin barrier proteins (filaggrin, occludin); inhibits JAK/STAT signaling pathway
Yang et al. (137)	2020	South Korea	hUC−MSCs	Ultracentrifugation	Lentiviral transduction for SOD3 overexpression	SOD3 protein	Regulates immune cell functions: suppresses T cell proliferation and Th1 differentiation, inhibits mast cell degranulation, enhances Treg generation	Subcutaneous injection	Reduces clinical symptoms, epidermal thickness, and lymphocyte/mast cell infiltration in DNCB-induced AD mouse model
Wang et al. (138)	2022	China	hUC−MSCs	Not specified in detail	None	Not specified in detail	Inhibits PBMC proliferation; promotes Treg differentiation; enhances endothelial tube formation (angiogenesis)	Subcutaneous injection	Accelerates wound closure; reduces skin lesion score and dermal lymphocyte infiltration; promotes neovascularization and collagen deposition; comparable to dexamethasone in healing efficacy
Zhao et al. (123)	2026	China	hUC−MSCs	Differential ultracentrifugation	None	Not specified in detail	Promotes exosome uptake by keratinocytes; reduces inflammatory cytokines (IL-5, IL-13, TSLP); alleviates oxidative stress and mitochondrial dysfunction; activates Wnt/β-catenin pathway	Topical application via thermosensitive poloxamer hydrogel	Reduces clinical symptoms (erythema, edema, SCORAD score); decreases serum IgE and Th2 cytokines (IL-4, IL-13, TNF-α, TSLP); improves epidermal hyperplasia and reduces mast cell infiltration; comparable efficacy to topical steroids with better safety profile
Kim et al. (139)	2026	South Korea	BMSCs	Size exclusion chromatography using LabSpinner™ system	Chemical hypoxic preconditioning with CoCl_2_	Enriched with cytokines, microRNAs, growth factors (e.g., via HIF-1α pathway), promoting angiogenesis, macrophage polarization, and barrier repair	Promotes HaCaT keratinocyte proliferation and migration; reduces inflammatory cytokines (IL-6, IL-33, CCL22, CCL17, MCP-1); increases IL-10 expression	Intradermal injection into mouse ears	Reduces ear thickness and epidermal hyperplasia; restores skin barrier proteins (filaggrin, involucrin, keratin 10); decreases immune cell infiltration (CD4+, CD45+, CD3+, F4/80+ cells); downregulates inflammatory cytokines and chemokines in oxazolone-induced AD model
Kim et al. (110)	2022	South Korea	iMSCs	Differential ultracentrifugation	IFN-γ priming	Enriched in interferon-response and inflammatory pathway proteins; contains CD63, CD81, TSG101	No cytotoxicity on human dermal fibroblasts and keratinocytes	Subcutaneous injection	Inhibits Th2 cytokine receptors (IL-4Rα/13Rα1/31Ra); reduces itching and skin inflammation; restores skin barrier and lipid synthesis in AD mice
Yoon et al. (140)	2023	South Korea	iMSCs	Ultracentrifugation	IFN-γ priming	Exosome markers (CD63, CD81, TSG101); enriched in IDO-related immunomodulatory molecules	Reduces TSLP, IL-25, IL-33 expression; increases KRT1, KRT10, DSG1, CerS3 expression in HaCaT cells	Epicutaneous and subcutaneous administration	Improves clinical scores, reduces TEWL, decreases epidermal/dermal thickness, reduces immune cell infiltration in Af-induced AD mice
Kim et al. (121)	2025	South Korea	iMSCs	Differential centrifugation + ultracentrifugation	IFN-γ priming	Not specified in detail	Suppresses IL-4/13-induced JAK1/2 and STAT6 signaling; reduces TSLP, IL-31, and itch receptor expression; restores KRT1 and filaggrin expression in keratinocytes	Subcutaneous injection	Reduces AD symptom scores, skin thickness, mast cell/inflammatory cell infiltration; suppresses Th2 cytokines and pruritus; restores skin barrier proteins; comparable or superior efficacy to baricitinib/clobetasol with better safety (no weight loss)

AD, Atopic Dermatitis; EVs, Extracellular Vesicles; ASCs, Adipose-derived mesenchymal stem cells; WJ−MSCs, Wharton’s jelly-derived mesenchymal stem cells; hUC−MSCs, Human umbilical cord mesenchymal stem cells; BMSCs, bone marrow-derived mesenchymal stem cells; IFN-γ-iMSCs, IFN-γ-primed induced pluripotent stem cell-derived mesenchymal stem cells; TSLP, Thymic Stromal Lymphopoietin; TEWL, Trans−epidermal water loss; SPHK1, Sphingosine Kinase 1; S1P, Sphingosine-1-Phosphate; DNCB, 2, 4-Dinitrochlorobenzene; PBMC, Peripheral Blood Mononuclear Cells.

#### 6.1.1 EVs derived from adipose-derived stem cells

Among the various MSC-derived EVs used for AD treatment, EVs derived from human ASCs, demonstrate particularly promising therapeutic potential due to their accessibility and unique functional contents. A core advantage of ASC-EVs in treating AD is their ability to directly and synergistically repair the compromised epidermal permeability barrier and modulate the aberrant immune response. The barrier repair function is primarily achieved through regulating keratinocyte lipid metabolism. Research confirms that ASC-EVs themselves are enriched with barrier lipid precursors such as ceramides, sphingomyelins, and free fatty acids (141). More critically, they carry a unique “enzyme toolkit”: compared to parental ASCs, ASC-EVs exhibit higher activity of ceramide-synthesizing enzymes (e.g., acid sphingomyelinase) and sphingosine kinase 1 (SPHK1), while showing lower activity of ceramide-degrading enzymes (ceramidase) and sphingosine-1-phosphate (S1P)-degrading enzymes (S1P lyase/phosphatase) (133, 141). This unique enzymatic profile enables keratinocytes that have endocytosed ASC-EVs to effectively elevate intracellular levels of ceramide and its downstream metabolite S1P. The increase in ceramide directly promotes the formation and secretion of lamellar bodies, thereby repairing the stratum corneum lipid lamellar structure. This has been validated in an oxazolone-induced AD mouse model, manifested by significantly reduced transepidermal water loss and restoration of lamellar structures under electron microscopy (106). Concurrently, elevated S1P not only further promotes keratinocyte differentiation but also effectively inhibits the production of Th2-type inflammatory cytokines (e.g., IL-4, IL-13). The anti-inflammatory effects of ASC-EVs are attenuated by an SPHK1 inhibitor, suggesting a contributory role of the S1P signaling pathway in this process (106). This dual mechanism of “substrate replenishment and metabolic regulation” normalizes the epidermal barrier both structurally and functionally.

In terms of immunomodulation, ASC-EVs not only effectively reduced skin levels of Th2-related cytokines (IL-4, IL-5, IL-13) but also downregulated Th1-related cytokines (TNF-α, IFN-γ), whereas Dupilumab had limited impact on the Th1 pathway. More importantly, ASC-EVs demonstrated systemic immunomodulatory capacity: following subcutaneous injection, they could migrate to the spleen, restore the abnormal spleen CD4+/CD8+ T cell ratio in the AD model, and alleviate splenomegaly, indicating their ability to modulate the function of systemic immune organs (136). This pleiotropic effect also shows advantage in counteracting AD exacerbation triggered by environmental factors like particulate matter (PM). In a triple co-culture model of keratinocytes, fibroblasts, and mast cells simulating AD, ASC-EV treatment reversed PM-induced increases in pro-inflammatory cytokines (IL-6, IL-1β) and decreases in barrier protein (filaggrin, loricrin) expression, highlighting their protective role in complex inflammatory environments (132).

The clinical translational potential of ASC-EVs has received preliminary validation in addressing refractory clinical manifestations—particularly Dupilumab-associated facial erythema (DFR). DFR has heterogeneous etiologies and often shows poor response to conventional treatments. Clinical studies indicate that topical application of formulations containing ASC-EVs can rapidly and effectively alleviate DFR symptoms. In a prospective study, after five weeks of treatment, patients showed significant reductions in erythema index and clinical scores. Stratum corneum analysis revealed downregulation of inflammatory mediators (IL-1α, TSLP) and upregulation of barrier proteins (filaggrin) and repair factors (VEGF) (131). These findings are consistent with an earlier case report by Park et al., collectively confirming the ability of ASC-EVs to address clinical challenges through synergistic “anti-inflammatory + repair” actions (130). Furthermore, a case series study on moderate-to-severe facial AD indicated that topical use of an ASC-EV-containing cosmetic formulation significantly improved disease severity, skin hydration, transepidermal water loss, and pruritus, with a good safety profile (134), providing a basis for its use as an adjuvant or maintenance therapy. Additionally, ASC-EVs serve as an ideal engineering platform for potential precision therapy by loading specific functional molecules. For example, ASC-EVs enriched with miR-147a were associated with enhanced therapeutic effects in an AD model, concomitant with reduced expression of VEGFA and MEF2A-TSLP axis components (111). This provides proof-of-concept for developing next-generation targeted therapies based on EVs.

In summary, through their inherent lipid and enzyme supply, multi-pathway immunomodulatory capacity, and plasticity as drug carriers, ASC-EVs constitute an integrated therapeutic system targeting the vicious cycle of the “barrier-immune” axis in AD. From deep mechanisms (the ceramide-S1P metabolic axis) to systemic immunomodulation, and the preliminary resolution of refractory clinical issues, the existing evidence suggests the potential of ASC-EVs as a cell-free therapeutic strategy, pending further clinical validation. Notably, the dual Th1/Th2 suppressive and barrier-restorative profile of ASC-EVs positions them as particularly suited for Th2-high/extrinsic AD, mixed Th1/Th17-driven endogenous AD, and refractory presentations such as DFR (130, 131, 142, 143). Future research needs to focus on establishing large-scale, standardized production processes, identifying key active components, conducting large-scale randomized controlled clinical trials, and optimizing combination strategies with existing therapies to ultimately advance this innovative treatment towards clinical practice.

#### 6.1.2 EVs derived from umbilical cord mesenchymal stem cells

UC-MSC-EVs, as a class of highly promising cell-free therapeutic agents, offer a unique perspective and technological pathway for AD treatment that is distinct from adipose-derived EVs. Research in this area not only delves deeply into elucidating mechanisms of broad, multi-pathway immunomodulation but also has achieved significant progress in preparation processes, delivery strategies, and engineering modifications, collectively advancing the translation of this therapy towards clinical application.

At the level of therapeutic mechanisms, UC-MSC-EVs demonstrate comprehensive immunomodulatory capabilities that extend beyond single-target biologics. The research by Park et al. holds landmark significance. They employed an advanced 3D micro-pore sphere culture system for the large-scale production of Wharton’s Jelly (WJ)-MSC-EVs and conducted a direct comparison with the first-line biologic Dupilumab in an AD mouse model (135). The results showed that a single subcutaneous injection of EVs achieved comparable efficacy to repeated Dupilumab administration in improving dermatitis scores and repairing the skin barrier. However, a key distinction lies in the breadth of their action spectrum: while Dupilumab primarily and precisely inhibits the Th2 pathway, EVs simultaneously and effectively downregulated Th1-, Th2-, and Th17-related cytokines and significantly suppressed the expression of the key alarmin thymic stromal lymphopoietin (TSLP) (135). another comparative study in a house dust mite-induced AD animal model also showed that a single subcutaneous injection of clinically-scaled WJ-MSC-EVs was comparable to repeated Dupilumab injections in improving skin inflammation, but with a broader mechanism of action (136). This “multi-pronged” regulatory mode may offer greater advantage for AD, especially chronic or complex immune phenotypes, as it may help avoid compensatory Th1 pathway activation and related adverse effects potentially arising from the powerful single-target inhibition of Th2 (144). This effect has been associated with microRNA-146a carried by EVs, which has been shown *in vitro* to interact with IRAK1 and modulate the AKT/NF-κB signaling axis (135). Beyond modulating adaptive immunity, UC-MSC-EVs also promote tissue repair. Early work by Wang et al. confirmed their ability to accelerate wound healing in an eczema model, involving mechanisms such as inhibiting inflammatory infiltration, promoting regulatory T cell (Treg) conversion, and stimulating angiogenesis (138). Research by Shao et al. further focused the EVs’ action on a key early event in AD—neutrophil infiltration. They found that exosomes derived from hUC-MSCs were associated with reduced secretion of chemokines like CCL5 and CXCL11, concurrent with decreased STAT3 signaling in keratinocytes and diminished neutrophil recruitment to lesion sites (145). Additionally, Kim et al. elucidated a fine-tuned mechanism by which UC-MSCs and their secretome regulate another core component of AD—mast cell activation. They discovered that through the production of high levels of prostaglandin E2 (PGE2) and TGF-β1 in response to IL-4, they effectively inhibit mast cell degranulation and FcϵRI receptor expression (146). Collectively, these studies outline a clear picture of UC-MSC-EVs comprehensively intervening in the AD “itch-scratch-inflammation-barrier disruption” vicious cycle through synergistic, multi-target actions across multiple cell types.

To overcome translational bottlenecks such as low EV yield and limited *in vivo* delivery efficiency, researchers have made innovative breakthroughs in engineered preparation and novel delivery systems. Yang et al. adopted a genetic engineering strategy, preparing engineered EVs carrying the SOD3 protein by overexpressing extracellular superoxide dismutase 3 (SOD3) in human umbilical cord blood MSCs. These EVs not only inherited the enhanced antioxidant capacity of the parental cells but also delivered active SOD3 protein to target cells, demonstrating stronger anti-inflammatory and symptom-relieving effects in an AD model. This proves the feasibility of custom-enhancing EV functions by modifying parental cells (137). On the other hand, to address the poor targeting of systemic administration, Zhao et al. innovatively developed a thermosensitive poloxamer hydrogel for local delivery. This hydrogel can form an *in situ* gel on the skin surface at body temperature, enabling the sustained release and retention of hUC-MSC-exosomes (123). The exosome-loaded hydrogel showed significant efficacy in an AD model, comparable to the commonly used topical corticosteroid mometasone furoate, while avoiding potential systemic side effects (e.g., weight loss) associated with the latter. Mechanistic studies indicated that this therapy was associated with reduced oxidative stress and mitochondrial damage, concurrent with altered Wnt/β-catenin signaling in keratinocytes (123). The study by Park et al. represents an upstream innovation in preparation processes. Their system, combining 3D micro-pore sphere culture with tangential flow filtration, enables the large-scale, standardized production of EVs meeting clinical-grade requirements, significantly increasing yield and reducing batch-to-batch variation, thereby laying a material foundation for subsequent clinical studies (135).

In summary, EVs derived from UC-MSC, leveraging their inherent and broad immunomodulatory and tissue repair functions, enhanced efficacy through engineering modifications, improved local bioavailability via innovative delivery systems, and secured mass production and standardization through advanced preparation processes, have formed a complete R&D chain ranging from mechanistic exploration to clinical translation strategy design. These studies not only confirm the potential of UC-MSC-EVs as a therapy superior to or complementary to existing targeted treatments but also, by addressing key technical and theoretical issues in industrialization, are steadily advancing their translation from the laboratory towards becoming a new clinical treatment option for AD. Given their broad-spectrum suppression of Th1/Th2/Th17 pathways and favorable safety profile, UC-MSC-EVs appear particularly suited for chronic AD patients with mixed inflammatory endotypes [100-106]. Future research should continue to focus on large-scale GMP production, precise identification of active molecules, long-term safety evaluation, and efficacy validation in more diverse AD patient populations.

#### 6.1.3 EVs derived from bone marrow mesenchymal stem cells

In the exploration of cell-free therapeutic strategies for AD, EVs derived from human BM-MSCs have garnered significant interest due to their inheritance of the immunomodulatory and tissue repair functions of their parental cells. However, recent research frontiers indicate that engineering BM-MSCs through *in vitro* preconditioning can significantly enhance the therapeutic potency of their vesicles. The study by Kim et al. serves as a paradigm for this approach. They employed a chemical hypoxia preconditioning strategy using cobalt chloride (CoCl_2_) on MSCs to simulate a hypoxic environment and activate the intracellular hypoxia-inducible factor-1α (HIF-1α) signaling pathway (147, 148). This preconditioning strategy alters the cargo profile of exosomes secreted by the MSCs (termed Chem-exo), with increased levels of them with more active molecules related to angiogenesis, immunomodulation, and barrier repair (149), which may contribute to their observed effects.

Research confirms that these engineered EVs (Chem-exo) can synergistically target core pathological aspects of AD (139). *In vitro*, Chem-exo not only promotes the proliferation and migration of human keratinocytes (HaCaT), directly aiding epithelial regeneration, but also downregulates various key inflammatory cytokines such as IL-1β, IL-6, and IL-17A, breaking the inflammatory vicious cycle. In an oxazolone-induced chronic AD mouse model, the therapeutic efficacy of Chem-exo was comprehensively validated: it effectively reduced ear swelling, decreased dermal infiltration of immune cells like CD4^+^ and CD3^+^ T cells, and crucially restored the expression of core skin barrier proteins including filaggrin (FLG), involucrin (INV), and keratin 10 (K10) (150, 151). This dual action of promoting “inflammation suppression” and “barrier reconstruction” achieves a precise intervention on the dual pathological axes of AD.

The enhanced therapeutic effect likely stems from the “upgraded” vesicle cargo resulting from HIF-1α pathway activation, including the enrichment of microRNAs and anti-inflammatory mediators that promote macrophage polarization towards the M2 anti-inflammatory phenotype (152). From a translational perspective, this MSC-EVs therapy based on chemical preconditioning offers multiple advantages: it avoids the tumorigenicity and immunogenicity risks associated with live cell transplantation; its production process is simpler and more economical than traditional physical hypoxia culture; and it holds promise as a novel treatment option that is “steroid-sparing” and independent of biologics (153). These attributes position BM-MSC-EVs as a compelling therapeutic candidate for chronic AD patients presenting with profound epidermal barrier disruption, as well as those seeking sustained disease control while minimizing the long-term toxicity burden inherent to conventional systemic agents. Certainly, its clinical translation still faces common challenges such as EVs production standardization and *in vivo* pharmacokinetics (154). Nevertheless, the existing evidence clearly demonstrates that engineering MSCs through preconditioning is an effective strategy for unlocking the maximal therapeutic potential of their vesicles.

#### 6.1.4 EVs derived from induced mesenchymal stem cells derived from induced pluripotent stem cells

Within the therapeutic spectrum of EVs, iMSC-derived EVs represent an engineering pathway designed to fundamentally overcome core bottlenecks of traditional stem cell therapies, such as heterogeneity, limited expansion capacity, and tumorigenic risk. Their fundamental advantage lies in the ability to obtain theoretically limitless and highly homogeneous iMSCs from a single clone via iPSC reprogramming technology, providing an ideal “cell factory” for the large-scale, standardized production of therapeutic vesicles (155, 156). Crucially, studies consistently show that preconditioning iMSCs with inflammatory cytokines like IFN-γ significantly enhances the immunomodulatory cargo of their secreted vesicles, thereby demonstrating superior multi-target synergistic therapeutic potential in AD models (110, 121, 140).

The research by Soo Kim and Jimin Kim et al. systematically elucidates the mechanism of action and outstanding efficacy of these engineered EVs (IFN-γ-iMSC-EVs) (110, 121). Their core strength is the ability to synergistically intervene in three major pathological aspects of AD. In terms of suppressing excessive immune responses, IFN-γ-iMSC-EVs effectively downregulate the expression of key Th2 receptors (IL-4Rα, IL-13Rα1) and block the activation of their downstream JAK1/STAT6 signaling pathway, curbing the Th2-type inflammation that drives AD at its source (110, 121). Regarding itch relief, these vesicles significantly reduce levels of pruritogenic cytokines IL-31 and TSLP and their receptors, while inhibiting downstream phosphorylation of STAT1/STAT5, thereby interrupting the “itch-scratch” cycle (110, 121). Notably, in direct comparisons with existing standard therapies, IFN-γ-iMSC-EVs demonstrated efficacy comparable to or even superior to the JAK inhibitor baricitinib or the potent corticosteroid clobetasol propionate in reducing epidermal hyperplasia and immune cell infiltration, and were not associated with weight loss under the tested conditions (121). Proteomic analysis further revealed the material basis for this superiority: IFN-γ preconditioning enriches the vesicles with a protein network involved in interferon response and JAK/STAT signaling regulation, transforming them into a natural multi-target signaling “nanocomplex” (110). Whether these changes causally underlie the observed effects requires validation.

Research by Yoon et al. validates and complements these findings from another perspective (140). In an Aspergillus fumigatus-induced AD model, EVs derived from IFN-γ-preconditioned iMSCs, IFN-γ-iMSC-EVs, effectively alleviated symptoms via either topical or subcutaneous administration. The mechanism similarly involved a two-pronged approach: on one hand, inhibiting the production of epithelial alarmins like TSLP and IL-25, and on the other hand, directly upregulating the expression of keratinocyte differentiation markers (keratin KRT1, KRT10) and ceramide synthase 3 (CerS3), thereby synergistically promoting skin barrier repair (140). This study also highlights the unique advantages of iMSC-EVs as cell-free products: compared to their parental cells, EVs are easier for local administration, avoid non-targeted accumulation in organs like the lungs and liver, carry no tumorigenic risk, and are more suitable for storage and quality control (140).

In summary, iMSC-EVs, particularly after engineering enhancement via IFN-γ preconditioning, successfully combine the “pleiotropic” benefits of stem cell therapy with the advantages of cell engineering: “standardization and scalability.” By synergistically inhibiting Th2 immunity, blocking neuro-itch signaling, and directly promoting barrier repair, they achieve integrated intervention across multiple pathological pathways in AD. Based on these properties, iMSC-EVs may be particularly suited for AD patients with predominant pruritus or Th2-driven endotypes, and could represent a promising option where standardized, off-the-shelf cell-free formulations are clinically prioritized. This offers a highly promising new strategy for developing next-generation, highly effective, homogeneous, and safer “off-the-shelf” cell-free therapeutic products. Nevertheless, several limitations should be acknowledged. First, while IFN−γ preconditioning enhanced therapeutic efficacy, the detailed molecular mechanisms by which these engineered vesicles negatively regulate interleukin receptors and their downstream signaling remain to be fully elucidated. Second, the long−term safety and potential off−target effects of iMSC−EVs have not been systematically evaluated, and the key bioactive components responsible for their multi−target actions are yet to be identified. Third, as with most preclinical studies, the DNCB− or Aspergillus−induced AD models do not fully recapitulate the chronic, relapsing, and heterogeneous nature of human AD, warranting caution when extrapolating the findings to clinical settings.

### 6.2 EVs derived from other somatic cells and engineered cell sources

#### 6.2.1 EVs derived from neural stem cells

NSCs and their secreted vesicles (NSC-EVs), a previously underappreciated stem cell source, are demonstrating unique therapeutic promise. Research by Lee et al. provides the first systematic evaluation of the potential of NSC-EVs in an AD model (122). *In vitro*, NSC-EVs were effectively taken up by keratinocytes and, by inhibiting the key NF-κB inflammatory signaling pathway, significantly downregulated the expression of various pro-inflammatory cytokines such as IL-6 and TNF-α, as well as inducible nitric oxide synthase (iNOS). In a DNCB-induced AD mouse model, topical application of NSC-EVs effectively alleviated skin inflammation, epidermal hyperplasia, and mast cell infiltration, visibly improving both the clinical and histopathological symptoms of AD.

In-depth proteomic analysis conducted in this study offers crucial insights into the mechanism of action (122). The analysis revealed that NSC-EVs are specifically enriched with two major categories of functional proteins: one category consists of proteins closely related to extracellular matrix (ECM) remodeling and active repair, such as fibronectin (FN1) and type I collagen (COL1A1/A2) (157, 158); the other category includes proteins with potent immunomodulatory and anti-inflammatory potential, such as SERPINA1 and alpha-2-macroglobulin (A2M) (159, 160). This unique cargo composition suggests that the action of NSC-EVs extends beyond mere anti-inflammatory suppression. Instead, they synergistically deliver both “repair signals” and “regulatory signals, “ actively promoting the reconstruction of skin structure and restoration of homeostasis while inhibiting the excessive immune response. This provides a molecular basis for explaining their dual efficacy in the model, which included both reducing inflammation and improving barrier integrity.

In conclusion, EVs derived from neural stem cells, distinguished by their unique proteomic signature, demonstrate the potential to intervene in AD pathology through synergistic multi-target actions. Given their dual capacity for anti-inflammatory modulation and tissue remodeling, NSC-EVs may hold particular relevance for AD cases requiring concurrent management of inflammation and structural repair. Although their precise network of action awaits full elucidation, the existing evidence already establishes them as a promising and novel source worthy of exploration within the field of cell-free therapy for AD.

#### 6.2.2 EVs derived from dermal fibroblasts

DF are the primary cells in the skin’s connective tissue, responsible for the synthesis and remodeling of the ECM, and are crucial for maintaining skin homeostasis. Exosomes isolated from the conditioned medium of DF (such as HDFn-Ex) are rich in bioactive substances like proteins, lipids, and nucleic acids. They can be effectively taken up by target cells like keratinocytes, thereby modulating their function. Studies show that HDFn-Ex can significantly upregulate the expression of peroxisome proliferator-activated receptor alpha (PPARα), a key nuclear receptor regulating epidermal differentiation, lipid synthesis, and barrier function (161, 162). In a DNCB-induced *in vitro* AD model, HDFn-Ex effectively reversed the downregulation of key barrier proteins—filaggrin (FLG), involucrin (IVL), loricrin (LOR), and hyaluronic acid synthases 1/2 (HAS1/HAS2)—caused by DNCB, through the activation of PPARα. These protective effects could be blocked by the PPARα-specific antagonist GW6471, supporting a contributory role of the PPARα pathway (163). This aligns with previous research showing that PPARα agonists (e.g., Wy14643) can promote ceramide synthesis and accelerate barrier maturation (164).

Beyond directly enhancing barrier protein expression, HDFn-Ex also exerts comprehensive therapeutic effects by modulating downstream signaling pathways. In AD, the mitogen-activated protein kinase (MAPK) signaling pathway is aberrantly activated, contributing to inflammation and barrier disruption. HDFn-Ex treatment inhibits DNCB-induced MAPK pathway activation, an effect correlated with its ability to restore FLG expression, suggesting the existence of an “FLG-MAPK” feedback regulatory axis (165, 166). Furthermore, HDFn-Ex exhibits potent anti-inflammatory properties. In a DNCB-induced AD-like mouse model, topical application of HDFn-Ex not only improved epidermal hyperplasia, hyperkeratosis, and transepidermal water loss but also significantly reduced dermal mast cell infiltration as well as serum levels of IgE and IL-4 (163). At the molecular level, HDFn-Ex inhibited DNCB-induced IκBα phosphorylation and subsequent nuclear translocation of NF-κB, thereby reducing the expression of key pro-inflammatory factors like TNF-α. This anti-inflammatory effect also depends on PPARα activation (167, 168).

Compared to currently used topical corticosteroids or calcineurin inhibitors, EVs derived from dermal fibroblasts offer potential advantages such as high stability, low immunogenicity, the ability to directly stimulate target cells, and the absence of side effects like skin atrophy associated with long-term steroid use (169, 170). Relative to extensively studied MSC-derived EVs, DF-EVs present unique benefits in treating skin disorders: fibroblasts are more easily obtained and cultured from skin tissue, their origin is closer to the target tissue, and they circumvent the invasive procedures and potential contamination risks associated with MSC extraction (109, 171). Further research has optimized treatment parameters, finding that a concentration of 1×10^4^ particles/mL of DF-EVs was most effective and minimally cytotoxic in restoring barrier protein expression in a HaCaT cell model, providing an important reference for clinical dosage exploration (172). Taken together, these characteristics suggest that DF-EVs may be particularly applicable to AD patients with prominent barrier dysfunction or those who are intolerant to long-term corticosteroid use.

#### 6.2.3 Nanovesicles from human embryonic kidney -293 cells

The HEK293 cell line is highly favored for its ease of transfection, rapid proliferation, and well-established platform for recombinant protein production, making it an ideal “cell factory” for generating customized EVs and, in particular, nanovesicles (NVs). An innovative study by Kim et al. cleverly leveraged this platform by physically extruding HEK293 cells while simultaneously loading them with melatonin, producing EV-mimetic “melatonin-loaded nanovesicles” (MelaNVs). This successfully constructed a novel and efficient transdermal delivery and therapeutic system (173).

The core advantage of this strategy lies in the synergistic integration of the efficient delivery capability of engineered NVs with the pleiotropic therapeutic potential of melatonin. On one hand, the MelaNVs prepared via extrusion (average size ~100 nm) inherit the biocompatibility and deep penetration potential of cell membrane-derived vesicular structures, effectively overcoming the bottleneck of melatonin’s inherently low transdermal efficiency (173, 174). On the other hand, the loaded melatonin is an endogenous hormone with potent antioxidant and anti-inflammatory properties. Previous studies suggest its ability to protect keratinocytes, inhibit Th2-type immune responses, and suppress mast cell activation (175–177). Experiments by Kim et al. confirmed that this integration produces a significant synergistic effect: *in vitro*, MelaNVs were more effective than an equivalent concentration of free melatonin in inhibiting LPS-induced TNF-α production in macrophages and compound 48/80-induced mast cell degranulation (173). In a DNCB-induced AD-like mouse model, topical application of MelaNVs (at a dose equivalent to only 1 mg/kg melatonin) demonstrated robust comprehensive therapeutic effects, including: significantly reducing epidermal hyperplasia and dermal inflammatory cell (especially mast cell) infiltration; lowering total serum IgE levels and modulating the IFN-γ/IL-4 balance; inhibiting the expression of pro-inflammatory mediators like cyclooxygenase-2 (COX2) and TNF-α in lesions; and, importantly, downregulating the expression of protease-activated receptor 2 (PAR-2) (173). PAR-2 plays a key role in AD pruritus, skin barrier disruption, and Th2 immune skewing; its downregulation provides a molecular explanation for part of MelaNVs’ mechanisms in alleviating itching and repairing the barrier (178, 179).

Notably, the study also revealed an insightful finding: even HEK293-NVs without drug loading exhibited a certain degree of anti-inflammatory and barrier-protective activity *in vivo* (173). This suggests that vesicles derived from HEK293 cells themselves may carry inherent bioactive substances (e.g., proteins or nucleic acids) capable of modulating the skin microenvironment. This finding resonates with other research, such as EV-mimetics prepared from HEK293 cells overexpressing specific genes promoting diabetic wound healing (180), and mesenchymal stem cell-derived NVs also demonstrating independent anti-inflammatory properties (181). Therefore, HEK293-derived EVs/NVs can be regarded as a versatile platform whose therapeutic effects stem from the superposition and synergy between the “intrinsic activity of the carrier itself” and the “therapeutic cargo it carries.” In this context, HEK293-MelaNVs may be particularly relevant for AD presentations in which PAR-2-mediated pruritus is a predominant feature (**Table 2**).

**Table 2:** Summary of study characteristics and outcomes for Non-MSCs derived EVs in AD.

Author	Year	Country	EVs source	Isolation methods	Modification	Cargoes	Outcome (*in vitro* or ex vivo)	*In vivo* (delivery route)	Outcome (*in vivo*)
Lee et al. (122)	2025	South Korea	NSCs	Centrifugation + 0.22 μm filtration + Amicon Ultra 100K + EXo-I column purification	None	Immune-regulatory proteins (S100A8, SERPINA1, A2M); ECM-remodeling proteins (FN1, COL1A1, COL1A2)	Reduces proinflammatory cytokines and chemokines; inhibits NF-κB phosphorylation; suppresses macrophage activation	Topical application	Alleviates AD symptoms; reduces mast cell infiltration and epidermal thickness; improves skin barrier integrity in DNCB-induced AD mice
Yoo et al. (172)	2023	South Korea	Dermal fibroblasts	Differential ultracentrifugation	None	Not specified in detail	At optimal concentration, promotes expression of skin barrier proteins (FLG, IVL, LOR, HAS1) in HaCaT cells; restores expression of these proteins in a DNCB-induced AD cell model	Not performed	Not performed
Jang et al. (163)	2024	South Korea	Human neonatal dermal fibroblasts	Ultracentrifugation + Tangential flow filtration	None	Contains exosomal markers (CD63, ALIX); rich in proteins and RNAs involved in skin barrier and anti−inflammatory regulation	Increases PPARα expression; restores filaggrin, involucrin, loricrin, HAS1, HAS2 levels; suppresses DNCB−induced ROS and MAPK pathway activation in HaCaT cells; improves epidermal morphology in 3D skin model	Intraperitoneal injection	Improves dermatitis score; reduces epidermal thickening and mast cell infiltration; decreases TEWL and increases skin hydration; lowers serum IgE and IL−4 levels; downregulates TNF−α expression in DNCB−induced AD mouse model
Kim et al. (173)	2021	South Korea	HEK293 cell	Extrusion through polycarbonate membranes + density gradient ultracentrifugation	Loaded with melatonin	Contains EV markers (CD81, CD9); melatonin (~97.1 ng/μg protein)	Inhibits TNF-α release in LPS-stimulated RAW264.7 cells; suppresses β-hexosaminidase in C48/80-treated RBL-2H3 cells; more effective than free melatonin	Topical application	Reduces DNCB-induced AD-like skin symptoms (erythema, edema, epidermal thickening); decreases mast cell infiltration & collagen deposition; lowers serum IgE; modulates IFN-γ/IL-4 balance; suppresses COX-2, TNF-α, and PAR-2 expression in skin
Huang et al. (182)	2023	China	Grapefruit-derived exosome-like nanovesicles + CCR6-NVs	GEVs: Differential centrifugation + sucrose gradient ultracentrifugation; CCR6-NVs: Membrane extrusion from engineered GMSCs	Fusion of CX5461-loaded GEVs with CCR6-NVs via membrane extrusion to form FV@CX5461	CX5461 (immunosuppressant); plant miRNAs (e.g., csi-miR159a); lipids (ceramides, hexosylceramides); flavonoids	Anti-inflammatory and antioxidant effects; inhibited T cell proliferation and migration; promoted M2 macrophage polarization; downregulated JAK-STAT and TCR signaling pathways	Intravenous injection	Targeted inflamed skin via CCR6–CCL20 axis; alleviated psoriasis and AD symptoms; reduced inflammatory cell infiltration and cytokines; increased Tregs; enhanced epidermal barrier; safe and biocompatible
Long et al. (183)	2025	China	Portulaca oleracea L. (purslane)	Differential centrifugation + ultracentrifugation	None	Proteins (<95 kDa) and mRNAs; transcriptomics revealed immune-related genes	Inhibits TNF-α-induced HaCaT cell proliferation; suppresses LPS-induced NO, TNF-α, and IL-6 in RAW264.7 cells; promotes macrophage polarization from M1 to M2 phenotype	Dissolvable microneedle patch (hyaluronic acid + purslane polysaccharides)	Ameliorates DNCB-induced AD symptoms: reduces skin thickening, erythema, mast cell infiltration; restores skin barrier function; downregulates TNF-α, IL-4, IgE; upregulates IFN-γ; inhibits NF-κB and STING signaling pathways; promotes M1-to-M2 macrophage shift in skin tissue
Zhong et al. (184)	2025	China	Turmeric-derived nanovesicles	Differential centrifugation + ultracentrifugation	Loaded into Fe³^+^-dopamine-modified hyaluronic acid hydrogel (Fe-HD@TDNV) for sustained release and antioxidative microenvironment	Rich in lipids, organic acids, organic nitrogen compounds; regulates pathways related to metabolism, inflammation, antioxidation, antibacterial activity, apoptosis	Enhances keratinocyte function (barrier proteins, antimicrobial peptides, antioxidant enzymes); reduces oxidative stress and DNA damage; promotes cell survival under H_2_O_2_-induced stress	Topical application as hydrogel dressing	Improves skin hydration; reduces epidermal thickness and mast cell infiltration; restores skin barrier proteins (filaggrin, involucrin, loricrin); enhances antimicrobial peptides; downregulates inflammatory cytokines; lowers serum IgE levels in DNCB-induced AD mice
Jo et al. (185)	2025	South Korea	Sea cucumber extracellular matrix	Enzymatic digestion (collagenase) + ExoQuick-TC	None	Diverse proteins and miRNAs (including 22-nt miRNAs)	Suppresses pro-inflammatory cytokines (TNF-α, IL-1β, IL-6, MCP-1, iNOS, NF-κB); upregulates anti-inflammatory factors (IκBα, SOCS-3) in LPS-induced macrophages	Subcutaneous injection	Reduces skin thickness, clinical dermatitis score, serum IgE levels, and mast cell infiltration in DNCB-induced AD mouse model
Wu et al. (183)	2024	China	Pinctada martensii mucus	Differential centrifugation + ultracentrifugation	None	Enriched in miR-100-5p; also contains CD9, CD63, CD81, HSP70, HSP90, etc.	Suppresses LPS-induced inflammation and pyroptosis in HaCaT cells; reduces ROS, LDH release, and lysosome damage	Topical application	Reduces skin inflammation, thickness, and inflammatory cell infiltration; restores collagen volume; downregulates NLRP3 inflammasome and inflammatory markers (iNOS, COX-2, IL-6, TNF-α) in DNFB-induced AD mice
Zhou et al. (186)	2025	China	Staphylococcus epidermidis (ATCC12228)	Ultracentrifugation	Loaded with Levofloxacin via freeze-anneal-thaw method	Levofloxacin, along with native EV components (proteins, lipids, nucleic acids)	Selective antibacterial activity against S. aureus; enhanced epidermal barrier function; modulated epidermal immune responses; promoted fibroblast proliferation and migration	Transdermal delivery via hyaluronic acid-based dissolving MN patch	Reduced skin inflammation, decreased S. aureus burden, enhanced dermal repair, induced IL-17A^+^ CD8^+^ T cells, improved epidermal barrier integrity in AD mouse model
Choi et al. (187)	2025	South Korea	Limosilactobacillus fermentum (SLAM216)	Super absorbent polymer beads + PEG precipitation + ultracentrifugation	None	Proteins (e.g., tnpA, yitT), lipids (AcylGlcADG, ceramides), fatty acids (linoleic, oleic, palmitic acids), miRNAs (e.g., bantam, mir-9-1)	Promotes wound healing in HaCaT cells; downregulates TSLP, TNF-α, IL-6, IL-1β, MDC; extends lifespan and enhances pathogen resistance in C. elegans	Oral administration	Reduces AD-like skin symptoms (redness, scaling, epidermal thickness, mast cell infiltration); decreases scratching, depressive/anxiety-like behaviors; modulates gut microbiota and increases serum serotonin levels in DNCB-induced AD mouse model
Li et al. (188)	2025	China	Limosilactobacillus reuteri (CCFM1040)	Ultracentrifugation after bacterial removal, followed by ultrafiltration and final ultracentrifugation	None	Enriched in aminoacyl tRNA synthetase-related proteins (e.g., Histidyl-tRNA synthetase); cytoplasmic proteins predominant	Promotes HaCaT cell migration; upregulates barrier proteins (Occludin, Claudin7, FLG, LOR); downregulates TSLP, TRPV1, TNF-α, IL-31RA, STAT3/p-STAT3; increases IL-10 in histamine-induced AD cell model	Oral administration (intragastric gavage)	Accumulates in skin; reduces epidermal thickness and mast cell infiltration; decreases serum IgE, histamine, IL-4, IL-13; upregulates skin FLG and LOR; inhibits skin TRPV1, TSLP, and STAT3/p-STAT3 in DNFB-induced AD mouse model
Mun et al. (189)	2025	South Korea	Bovine colostrum	Sequential ultracentrifugation	None	Not specified in detail	Not performed	Oral gavage (primary route; topical application also tested but less effective)	Alleviates skin lesions and ear thickness; reduces serum IgE, histamine, and inflammatory cytokines (IL-4, IL-13, TNF-α); decreases mast cell infiltration; restores skin barrier gene (Filaggrin) expression; modulates gut microbiota (increases Lactobacillus, Bifidobacterium); alters gut metabolome (e.g., increases lactic acid).

EVs: Extracellular Vesicles; NSCs; Neural stem cells; ECM: Extracellular matrix; HEK293: Human embryonic kidney 293 cells; MN: microneedle; CCR6-NVs: CCR6-engineered gingiva-derived mesenchymal stem cell membrane nanovesicles; TDNV: Turmeric-derived nanovesicles; DNFB: 2, 4-Dinitrofluorobenzene

### 6.3 EVs derived from plants

Plant-derived EVs, as a novel class of therapeutic agents, leverage the inherent biocompatibility, low immunogenicity, and rich repertoire of natural bioactive components (e.g., anti-inflammatory and antioxidant molecules) of their “green” origin. This provides a unique “active carrier” platform for AD treatment (190). Their prominent advantage lies in the ability to combine the pleiotropic therapeutic potential of natural products with the highly engineerable properties of nanocarriers. Through strategies like drug loading, membrane fusion modification, or integration into advanced delivery systems (e.g., microneedles, hydrogels), they aim to achieve multiple goals: efficacy enhancement, targeted delivery, and overcoming the transdermal barrier. This opens a new path for developing innovative therapies derived from nature yet superior to traditional ones (191) (**Figure 2**).

#### 6.3.1 EVs derived from grapefruit

EVs derived from grapefruit (GEVs) serve as a representative platform. They have been shown to possess inherent anti-inflammatory and antioxidant activity themselves (192), and their lipid bilayer structure further acts as a natural, “green” nanocarrier for efficiently encapsulating and delivering hydrophobic drug molecules (193). Research by Huang et al. (182) elevates the potential of this platform to a new level. They constructed an innovative engineered hybrid, FV@CX5461, based on a biohybrid strategy. This strategy first involves encapsulating the small-molecule drug CX5461—which has potent immunosuppressive and anti-proliferative activity (194)—within GEVs. To explore potential lesion-targeting capability, the research team performed membrane fusion between the drug-loaded GEVs and nanovesicles derived from genetically engineered gingival mesenchymal stem cells (GMSCs). The latter express the CCR6 chemokine receptor on their surface. This design was associated with the engineered hybrid distribution: First, it integrates the natural bioactive components of GEVs (such as flavonoids, terpenoids, and polysaccharides) with the direct inhibitory effects of CX5461 on abnormally activated T cells and keratinocytes (195, 196). Second, the CCR6 receptors embedded on the vesicle surface act as “molecular navigators, “ driving the active homing of the vesicles to the site of AD lesions where the chemokine CCL20 is highly expressed, suggesting potential for enhanced local delivery (182).

In an AD disease model, FV@CX5461 demonstrated outstanding multi-target synergistic therapeutic effects. It not only effectively reduced local levels of pro-inflammatory cytokines but, more importantly, could systematically reshape the disordered immune microenvironment: it significantly inhibited the activation of pathogenic Th17 cells while promoting the infiltration of immunomodulatory Tregs. This effect was significantly superior to traditional dexamethasone treatment (182). In-depth mechanistic analysis further revealed that the plant-derived bioactive molecules carried by GEVs themselves, particularly certain microRNAs (e.g., miR-159), have been detected in these vesicles (197). Whether such molecules can functionally regulate mammalian keratinocytes through cross-kingdom mechanisms remains to be experimentally validated.

In summary, the engineered hybrid strategy based on grapefruit-derived vesicles represents a cutting-edge paradigm that combines natural plant carriers, efficient synthetic drugs, and cellular engineering targeting technology. Given the dual advantages of natural origin and engineered targeting, this platform may be particularly suited for AD patients requiring long-term maintenance therapy or those with concerns regarding synthetic drug accumulation. It not only offers an innovative solution to overcome the bottlenecks of traditional therapies, such as significant side effects and lack of targeting (198, 199), but also opens new perspectives for understanding “plant-mammal” interactions by demonstrating the cross-kingdom regulatory potential of plant miRNAs.

#### 6.3.2 Nanovesicles derived from Portulaca oleracea

Nanovesicles derived from Portulaca oleracea (PDNVs) have garnered significant attention due to the long history of their parent plant’s use in traditional medicine for treating skin inflammation (200, 201). The research by Long et al. not only provides an in-depth explanation of PDNVs’ mechanism of action but also offers an important paradigm for addressing the clinical application bottlenecks of plant-derived vesicles through innovative formulation engineering (202).

Studies indicate that the core therapeutic mechanism of PDNVs lies in their potent immunomodulatory capacity. They exert anti-inflammatory effects in AD models by inhibiting key inflammatory signaling pathways such as NF-κB and the stimulator of interferon genes (STING), and by promoting the polarization of macrophages from the pro-inflammatory M1 phenotype to the anti-inflammatory M2 phenotype (202). This mechanism aligns with findings from other plant-derived vesicles, such as those from Pueraria lobata (kudzu) and garlic, which also alleviate inflammation by modulating macrophage function, highlighting the conserved potential of plant vesicles in immunomodulation (203, 204). However, plant-derived vesicles commonly face challenges such as heterogeneous size and poor transdermal permeability, which severely limit their efficacy (205). A key innovation of this study lies in proactively constructing an integrated solution combining “active therapy and efficient delivery.” The research team designed a novel soluble microneedle patch with a matrix composed of a composite of hyaluronic acid (HA) and Portulaca oleracea polysaccharide (POP), used to load and deliver PDNVs (202). This design offers a dual advantage: First, the incorporation of POP effectively mitigates the insufficient mechanical strength often seen in pure HA microneedles caused by high hygroscopicity, ensuring the microneedles can effectively penetrate the stratum corneum (206, 207). Second, POP itself may possess anti-inflammatory and antioxidant activities (207), potentially creating a synergistic therapeutic effect with PDNVs. This PDNV-loaded composite microneedle successfully achieved efficient transdermal delivery of the bioactive vesicles, resulting in significantly superior therapeutic outcomes compared to the topical application of PDNVs alone in an AD animal model.

In summary, this research goes beyond mere functional validation. By integrating naturally derived plant vesicles with well-defined immunomodulatory activity and a rationally designed advanced transdermal delivery system (the POP-HA composite microneedle), it provides a practical and feasible strategy for overcoming the translational challenges associated with plant-derived NVs. This minimally invasive, sustained-release formulation may be particularly advantageous for AD patients with poor adherence to daily topical regimens or those requiring prolonged therapeutic coverage.

#### 6.3.3 Nanovesicles derived from turmeric

Turmeric-derived nanovesicles (TDNVs), leveraging the long-standing tradition of their parent plant for anti-inflammatory and antioxidant properties, offer a highly innovative multi-target strategy for the root-cause treatment of AD—skin barrier repair. Research by Zhong et al. not only revealed the potent biological functions of TDNVs themselves but also achieved synergistic dual repair of AD barrier damage by constructing an intelligent “active vesicle-functional material” composite system (Fe-HD@TDNV) (184).

The core advantage of TDNVs lies in their ability to perform “triple synergistic repair” on the compromised skin barrier in AD. Studies confirm that TDNVs can simultaneously upregulate the expression of three major classes of key functional molecules in keratinocytes (184): First, structural barrier proteins such as filaggrin, loricrin, and involucrin, directly reconstructing the weakened physical barrier to combat the hallmark protein deficiency in AD (208, 209). Second, endogenous antimicrobial peptides, enhancing the skin’s intrinsic chemical defense and lowering the risk of secondary infections. Third, antioxidant enzymes (e.g., superoxide dismutase, catalase), boosting the cell’s intrinsic capacity to resist oxidative stress. This unique ability to act simultaneously on structural, immune, and metabolic dimensions enables TDNVs to comprehensively address the complex pathological network of AD.

Another significant contribution of this research is its ingenious design of a delivery and efficacy-enhancing system. The researchers constructed a metal-phenolic hydrogel (Fe-HD), formed by crosslinking Fe³^+^ with hyaluronic acid-dopamine, to serve as a carrier for TDNVs (184). This design goes far beyond simple physical loading: the Fe-HD hydrogel itself can actively create a “low oxidative stress” local microenvironment at the application site. This not only protects the activity of TDNVs but also synergizes with their antioxidant components, providing external “protection” for the repair process from the outside. Therefore, the Fe-HD@TDNV system constitutes an internally and externally coordinated “repair alliance”: internally, TDNVs directly empower keratinocytes, activating their regeneration and defense programs; externally, the Fe-HD hydrogel provides a protective microenvironment and ensures sustained release. In an AD model, this system significantly improved skin hydration, reduced epidermal hyperplasia, and suppressed abnormal immune responses, demonstrating marked therapeutic efficacy. Given this dual-action framework combining antioxidant protection and barrier restoration, the Fe-HD@TDNV system may hold particular promise for AD cases characterized by oxidative stress and impaired epidermal integrity.

### 6.4 EVs derived from marine organisms

EVs derived from marine sources are emerging as a novel class of therapeutic agents, opening a new exploratory direction for AD treatment with their unique bioactivity and origin advantages. The vast majority of current research focuses on terrestrial mammals, which constitute only about 6.2% of animal biomass, while the therapeutic potential of EVs from marine animals, representing the majority of biomass (77.2%), remains largely untapped (210) (**Figure 2**).

#### 6.4.1 EVs derived from sea cucumber

Recently, groundbreaking research by Han Jo et al. has, for the first time, focused on a marine echinoderm with exceptional regenerative and immunomodulatory capabilities—the sea cucumber. They successfully isolated and characterized anchored EVs from the sea cucumber’s ECM (185). To circumvent the issue of common bacterial contamination in seawater, the research team developed an optimized purification protocol based on enzymatic treatment to obtain high-purity EVs from the sea cucumber ECM. Physical characterization revealed that EVs derived from sea cucumber possess typical vesicular morphology and a size distribution (diameter ~30–200 nm) similar to mammalian exosomes. Despite interspecies differences, compositional analysis confirmed that EVs derived from sea cucumber encapsulate a rich array of bioactive molecules, including proteins (such as high-molecular-weight collagen) and small RNAs, indicating their potential as multifunctional therapeutic carriers (185).

In terms of mechanistic exploration, EVs derived from sea cucumber demonstrated potent anti-inflammatory efficacy. In a lipopolysaccharide (LPS)-induced macrophage inflammation model, EVs derived from sea cucumber treatment significantly downregulated the expression of pro-inflammatory mediators like inducible nitric oxide synthase (iNOS), while upregulating anti-inflammatory factors such as IκBα and SOCS-3 (185). To gain deeper insights into their mode of action, researchers performed microarray analysis. The results showed that EVs derived from sea cucumber treatment significantly suppressed the expression of inflammation-related genes (e.g., CCL4, SLFN4, IFI16) and upregulated cell-protective genes (e.g., MT2). Crucially, pathway enrichment analysis clearly identified the core mechanism of sea cucumber-derived EVs: the inhibition of the NOD-like receptor (NLR) signaling pathway. This pathway is a key component of the innate immune system, and its overactivation is closely linked to various autoimmune diseases, including AD (211, 212). EVs derived from sea cucumber specifically downregulated key molecules of the NLR pathway, such as the NLRP3 inflammasome and IFI16, which are overexpressed in AD lesions, thereby potentially blocking the inflammatory cascade driven by this pathway (185).

This mechanism was effectively validated *in vivo* in an AD model. In a DNCB-induced mouse AD model, subcutaneous injection of EVs derived from sea cucumber significantly improved clinical disease manifestations, including lowering AD severity scores, reducing epidermal thickening, and decreasing dermal mast cell infiltration (185). Notably, EVs derived from sea cucumber treatment also significantly reduced serum levels of total IgE, a key marker of immune dysregulation in AD. Encouragingly, even at lower doses, EVs derived from sea cucumber exhibited a therapeutic trend comparable to higher doses, suggesting high bioactivity and a potential safety window. In summary, anchored EVs derived from sea cucumber ECM effectively alleviated skin inflammation, repaired barrier function, and modulated allergic immune responses in an AD model by inhibiting the critical NLR inflammatory signaling pathway. Given these immunomodulatory effects, sea cucumber-derived EVs may be particularly relevant for AD endotypes driven by type 2 immune dysregulation and IgE-mediated hypersensitivity. This study not only provides the first evidence for the feasibility of using marine animal-derived EVs to treat AD but also expands the source of EVs for therapeutic applications to the vast resources of marine organisms. It offers revolutionary concepts and solid experimental evidence for developing novel, effective, and safe cell-free therapeutic strategies derived from the ocean (185).

#### 6.4.2 EVs derived from Pinctada fucata martensii

Beyond echinoderms, marine bivalves also offer a unique source of EVs for AD treatment. A series of studies by Wu et al. systematically revealed the excellent therapeutic potential and intricate molecular mechanisms of EVs extracted from the mucus of Pinctada fucata martensii (PmEVs), a byproduct of pearl production (183, 213). The significance of this work lies not only in validating the efficacy of EVs from a low-cost, readily available marine biomaterial source but also in providing a preliminary framework for the potential future application of marine-derived EVs through delivery engineering and in-depth mechanistic analysis, though these findings remain preclinical and model-dependent.

The research team first confirmed the inherent anti-inflammatory efficacy of PmEVs, finding that they could significantly alleviate LPS-induced keratinocyte inflammation and symptoms in an AD mouse model by inhibiting the MAPK/NF-κB/NLRP3 signaling pathway (213). To elucidate their active material basis, they performed miRNA sequencing analysis on PmEVs and identified highly expressed miR-100-5p as one of their key functional components (183). However, the efficacy of locally administered EVs is often limited by their rapid clearance. To address this, the study innovatively developed an oxidized sodium alginate-carboxymethyl chitosan (OSA-CMCS) self-crosslinking hydrogel as a delivery vehicle. This hydrogel efficiently loaded PmEVs, significantly enhancing their retention at the lesion sites in AD mice and enabling sustained release for long-term treatment (183).

*In vivo* experiments confirmed that the PmEV-loaded hydrogel effectively reduced inflammation, lowered disease scores, and promoted dermal collagen synthesis in a DNCB-induced BALB/c mouse AD model, thereby contributing to the structural repair of the skin barrier. The breakthrough finding of the study lies in precisely elucidating its core molecular mechanism: the miR-100-5p delivered by PmEVs can target and inhibit the mRNA expression of the host cell transcription factor FOXO3. The downregulation of FOXO3, in turn, blocks the activation of its downstream NOD-like receptor protein 3 (NLRP3) inflammasome (183). The NLRP3 inflammasome is a core molecular platform driving the maturation and release of key pro-inflammatory cytokines like IL-1β, and its activation is crucial in AD pathology (214). Therefore, this study is the first to reveal a marine-derived EV-specific miRNA-mediated anti-inflammatory signaling axis: the miR-100-5p/FOXO3/NLRP3 axis. From a clinical translation perspective, this well-defined mechanistic axis suggests that PmEV-based therapy may be most beneficial for AD patients exhibiting pronounced NLRP3-driven neuroinflammatory features, such as those with severe pruritus and elevated IL-1β-associated inflammatory signatures.

### 6.5 EVs derived from microorganisms

Microbial-derived EVs offer a novel paradigm for AD treatment that transcends traditional antibiotics or simple anti-inflammatory strategies. Their core advantage lies in the ability to ingeniously harness the inherent biological properties of host-microbe interactions (e.g., with commensals or probiotics). They can be engineered into “biomimetic nanoplatforms” for precise drug delivery or used to systemically modulate the “gut-skin axis” and even the “gut-skin-brain axis” via oral administration. This approach enables the synergistic combination of local and systemic interventions, aiming to clear pathogens, repair the barrier, and modulate immunity while simultaneously preserving or reshaping microbiome homeostasis (215) (**Figure 2**).

#### 6.5.1 EVs derived from *Staphylococcus epidermidis*

Among microbial-derived EV strategies for AD, a highly innovative advancement focuses on utilizing vesicles from the skin commensal bacterium *Staphylococcus epidermidis* as an intelligent nanoplatform to address the clinically challenging issue of bacterially infected AD (186). This form of AD is difficult to cure due to epidermal barrier defects and the colonization and deep-seated infection by *S. aureus*. Traditional combination therapies of antibiotics and immunosuppressants often carry risks such as microbiome disruption and antibiotic resistance (216, 217).

SE-EVs themselves inherit the beneficial properties of their parent bacteria, being able to induce keratinocytes to secrete antimicrobial peptides and reduce inflammation. Their inability to replicate makes them safer than using live bacteria (218, 219). However, their inherent limitations include a lack of potent intrinsic antibacterial activity and difficulty penetrating thickened AD skin to reach sites of dermal infection (219, 220). Research by Zhou et al. (186) successfully overcame these bottlenecks through a two-step strategy: First, they efficiently loaded the antibiotic levofloxacin into SE-EVs using a freeze-thaw method, constructing the “nano-antibiotic” Lev@SE-EVs. This engineered vesicle not only retained the immunomodulatory and barrier-enhancing functions of SE-EVs but also acquired potent bactericidal capability. Crucially, as vesicles might be preferentially taken up by the related pathogen *S. aureus*, Lev@SE-EVs demonstrated the potential for selective killing of the pathogen, minimizing impact on the beneficial skin commensal flora (186, 221).

Second, to address the transdermal delivery challenge, the study integrated Lev@SE-EVs into a HA soluble microneedle patch, forming the Lev@SE-EVs@MN composite system (186). The microneedles painlessly penetrate the stratum corneum, efficiently and directly delivering Lev@SE-EVs to the S. aureus-rich dermis. In a mouse model of infected AD, this system achieved synergistic, multi-faceted therapeutic effects: significantly reducing dermal bacterial load, alleviating inflammatory cell infiltration, and promoting tissue repair. An important mechanistic discovery was that SE-EVs and their drug-loaded derivatives could induce the accumulation of protective IL-17A+ CD8+ T cells in the skin in a non-inflammatory-dependent manner (186, 219). This resembles the long-term immune surveillance provided by S. epidermidis colonization, potentially helping to prevent recurrence. This multi-pronged strategy may be particularly suited for AD patients with confirmed Staphylococcus aureus colonization.

In summary, this research demonstrates a precision treatment paradigm that combines “biomimicry” and “engineering.” It skillfully integrates the friendly immune signals of a commensal bacterium (delivered via its EVs), the targeted bactericidal action of an antibiotic, and the deep delivery capability of an advanced formulation. This approach concurrently addresses multiple clinical challenges: clearing pathogens, controlling inflammation, repairing the barrier, and maintaining microbiome homeostasis, providing a novel concept and strong experimental basis for treating complex infectious skin diseases. Despite these advances, several limitations should be acknowledged. The freeze-thaw loading method, while effective, altered EV particle size and polydispersity index, and its potential impact on EV functionality requires further investigation. Moreover, whether microneedle-assisted delivery offers superior epidermal effects and how exactly barrier restoration occurs in the AD model remain unclear. The mechanisms governing the preferential uptake of SE-EVs by S. aureus also warrant deeper exploration.

#### 6.5.2 EVs derived from Limosilactobacillus fermentum

Among probiotic-derived EVs, vesicles derived from *Limosilactobacillus fermentum* SLAM 216 (LF216EV) demonstrate unique potential that extends beyond traditional topical anti-inflammatory action, offering systemic intervention in AD by modulating the “gut-skin-brain” axis. Research by Choi et al. provides multi-layered evidence supporting this (187). The study first clarified the active material basis of LF216EV through multi-omics analysis, revealing its enrichment with specific lipids (such as ceramides and acylglucosyldiacylglycerol) and metabolites (such as linoleic acid), components inherently possessing anti-inflammatory and skin barrier-promoting potential (222, 223).

In a DNCB-induced AD mouse model, LF216EV treatment effectively alleviated skin erythema, epidermal hyperplasia, and dermal mast cell infiltration, visibly improving the cutaneous phenotype of AD (187). However, the standout contribution of this research lies in uncovering a more systemic and profound mechanism of action for LF216EV. The study found that oral administration of LF216EV significantly reshaped the gut microbiota of AD mice, increasing the abundance of beneficial bacteria such as the *Limosilactobacillus genus*, while concurrently elevating serotonin levels in both the gut and serum (187). This increase in serotonin levels was not only associated with reduced skin itch but, more critically, it directly mediated the rapid improvement of comorbid depressive- and anxiety-like behaviors in the AD model mice. Notably, the onset of this behavioral improvement was even faster than that observed with dexamethasone treatment (187). Mechanistically, this effect is likely linked to LF216EV’s modulation of the gut metabolome, promoting the shift of tryptophan metabolism toward the serotonin synthesis pathway (224, 225).

Therefore, EVs derived from *Limosilactobacillus fermentum* represent an innovative class of “bio-psycho-dermatological” integrated therapeutic agents. They act directly on local inflammation through their inherent active components while simultaneously alleviating AD-related skin inflammation, pruritus, and comorbid neuropsychiatric symptoms at a systemic level by modulating gut microbiota homeostasis and host serotonin metabolism. Consequently, oral Limosilactobacillus fermentum LF216 EVs may hold particular promise for AD patients with comorbid depression and anxiety. This provides a novel perspective and robust experimental foundation for developing next-generation oral therapies based on probiotic vesicles that can simultaneously target both the physiological and psychopathological dimensions of AD.

#### 6.5.3 EVs derived from *Limosilactobacillus reuteri*

Lr-EVs represent a significant research direction for treating AD through oral intervention targeting the “gut-skin axis.” Research by Li et al. systematically elucidates the therapeutic potential and precise mechanisms of orally administered EVs derived from *Limosilactobacillus reuteri* CCFM1040 (Lr1040-EVs) (188).

The study demonstrates that oral administration of Lr1040-EVs effectively alleviates symptoms in a DNFB-induced AD mouse model, including reducing epidermal hyperplasia, lowering serum IgE and histamine levels, and decreasing dermal mast cell infiltration. The core mechanism lies in the precise modulation of key pathological signaling networks in AD, particularly the STAT3 pathway (188). At the molecular level, Lr1040-EVs significantly upregulate the expression of tight junction proteins (Occludin, Claudin-7) and differentiation proteins (filaggrin, loricrin) in keratinocytes, directly reinforcing the skin’s physical barrier. Concurrently, by inhibiting the activation of the STAT3 signaling pathway, they effectively downregulate the expression of the key epithelial-derived alarmin TSLP, as well as core mediators of itch perception such as the TRPV1 ion channel and the IL-31 receptor (188). This dual inhibition of the “TSLP-STAT3” axis and the neuroimmune itch pathway forms the molecular basis of their potent anti-inflammatory and antipruritic effects.

In-depth proteomic analysis further revealed the functional material basis of Lr1040-EVs, showing that the vesicles are enriched with various aminoacyl-tRNA synthetases. Among these, histidyl-tRNA synthetase was identified as a key functional component (188). The researchers speculate that this enzyme may play a central role in alleviating inflammation and pruritus by mimicking or interfering with downstream signaling of histamine receptors (e.g., H1R), thereby inhibiting STAT3 pathway activation. Given the central involvement of the STAT3-TSLP axis and histamine receptor signaling in pruritus-driven AD pathogenesis, Lr1040-EVs may hold particular promise for this clinical endotype. This provides a new molecular perspective for understanding the cross-kingdom action of probiotic-derived vesicles.

### 6.6 EVs derived from bovine colostrum

BCEVs, as a type of natural bioactive EVs suitable for oral administration, demonstrate unique therapeutic potential. Research by Daye Mun et al. provides key evidence for this (189). In a DNCB-induced AD mouse model, oral administration of BCEVs effectively alleviated skin lesion severity, lowered serum IgE levels, and corrected the imbalance between the Th2-type cytokine IL-4 and the pro-inflammatory factor TNF-α, indicating systemic anti-inflammatory and anti-allergic effects (**Figure 2**).

The core mechanism of this therapy lies in its systemic remodeling of the gut microecology. Gut microbiota analysis via 16S rRNA gene sequencing revealed that BCEVs significantly reversed the AD-associated gut microbiota dysbiosis, specifically increasing the abundance of recognized beneficial bacteria such as *Lactobacillus* and *Bifidobacterium genera* (189). This prebiotic-like regulatory effect aligns with previous reports that milk-derived vesicles can modulate gut microbiota composition (226, 227). Simultaneously, metabolomic analysis further revealed that BCEV treatment elevated the levels of microbial metabolites, like lactate, in the gut contents (189). These changes collectively point towards an improved gut microenvironment. This synergistic regulation of gut microbiota and their metabolites may indirectly improve skin symptoms through various downstream pathways: the restoration of beneficial bacteria helps enhance intestinal barrier integrity, reducing endotoxin translocation and the consequent systemic low-grade inflammation (228, 229); metabolites like lactate may be involved in modulating local immunity or intestinal pH homeostasis; furthermore, active components carried by BCEVs themselves, such as miRNAs and proteins, might also exert immunomodulatory effects, either directly or by influencing the differentiation and function of host immune cells (e.g., regulatory T cells) (230, 231). Therefore, the action of BCEVs embodies an “inside-out” systemic therapeutic logic, whereby repairing this distal ecological niche—the gut—alleviates the localized immune inflammation in the skin. This gut-centric mechanism suggests that oral BCEVs may be particularly beneficial for AD patients with dysbiotic gut microbiota profiles, a subset increasingly recognized as the “gut dysbiosis-driven” AD endotype. Nevertheless, several limitations should be noted. The gut microbiota analysis provided correlative rather than causal evidence for the gut−skin axis, and the stability of BCEVs in the gastrointestinal tract as well as their oral bioavailability were not thoroughly characterized.

## 7 Current limitations and challenges of research and key directions for future studies

Although EV-based AD treatment research shows encouraging prospects, the field as a whole remains in the early stages of advancing from proof-of-concept towards clinical translation, facing a series of interrelated scientific, technical, and regulatory challenges. A deep analysis of these limitations and a clear definition of future research directions are crucial for maturing EV-based therapies.

Current research exhibits a significant imbalance and exploratory nature in EV source selection and engineering strategies. More than half of preclinical studies focus on ASC-EVs, driven by their good accessibility, validated safety profile, and clear immunomodulatory capabilities (127). However, EVs from other highly promising sources, such as umbilical cord or iPSC-derived MSCs, are relatively understudied, while sources like bone marrow or amniotic fluid are rarely explored. This skewed focus, although consistent with early trends in AD cell therapy, undoubtedly limits the search for the optimal therapeutic source (127). Concurrently, engineering strategies to enhance efficacy—such as preconditioning parental cells (e.g., with hypoxia or inflammatory cytokines) or constructing hybrid vesicles (e.g., by fusing plant vesicles with engineered cell membranes)—are emerging and show synergistic potential (182). However, these modifications, while improving function, also increase the complexity of manufacturing processes and the uncertainty of regulatory approval. Therefore, systematically comparing the efficacy, safety, and industrial feasibility of EVs from different sources, including those with various engineering modifications, is an essential path to determining the best therapeutic candidates.

In the realm of EV preparation, characterization, and quality control, the lack of standardization is a core bottleneck hindering research reproducibility and result comparability. Although techniques like tangential flow filtration (TFF) are increasingly favored for their suitability for large-scale production (232, 233), significant differences in isolation and purification protocols persist across different laboratories. A more serious issue is that many studies fail to strictly adhere to the MISEV2023 guidelines released by the International Society for Extracellular Vesicles (ISEV) and relevant standards from the International Society for Cell & Gene Therapy (ISCT) (36, 234). Specific manifestations include incomplete characterization of EV preparations (e.g., particle count, protein concentration, specific markers), insufficient characterization of parental cells, and, most critically, a lack of adequate verification of preparation purity (235). The coexistence of contaminants (e.g., protein aggregates, non-vesicular structures) makes it difficult to definitively attribute observed biological effects to the EVs themselves (31). Therefore, in future research, mandatory and meticulous adherence to existing guidelines for EV production and characterization, along with the establishment of unified potency/functional assay biomarkers (236, 237), forms the cornerstone for enhancing the overall research quality and credibility of the entire field.

The elucidation of mechanisms of action often remains at the level of phenomenological description, lacking depth and systematicity. Existing studies confirm that EVs can downregulate key cytokines such as IL-4, IL-13, and TSLP and improve barrier protein expression, with effects sometimes comparable to corticosteroids (109). However, the precise molecular logic behind these effects—specifically, which “cargo” (e.g., specific miRNA, protein) carried by EVs, through which intracellular signaling pathways (e.g., JAK/STAT, NF-κB), and how they sequentially regulate the functional networks of target cells (keratinocytes, T cells, mast cells, etc.)—remains unclear (110, 140, 238). Furthermore, the interaction between EVs and the AD skin microbiome (239), as well as their potential role in regulating the “gut-skin axis, “ represent blank spots awaiting deeper exploration. Future efforts need to combine multi-omics analysis, gene editing, reporter systems, and other techniques to map the detailed “molecular map” of EV action in cell and animal models, and even in patient samples.

Existing preclinical disease models have inherent limitations in simulating the complexity and heterogeneity of human AD. Research predominantly relies on chemically induced rodent models such as DNCB and MC903. Although these models can replicate some AD features (e.g., Th2 inflammation, epidermal hyperplasia), they often fail to fully reproduce the chronic, multifactorial pathophysiological panorama of human AD, especially the different endotypes (e.g., Th1-, Th2-, Th17-, Th22-dominant) defined in recent years (240, 241). Variations in induction methods, animal strains, and gender differences among models further complicate result interpretation. Although some scholars have proposed minimum standards for an ideal AD model, consensus has yet to be formed. Developing more accurate models, such as humanized skin mice, organoid models, or models focusing on simulating specific endotypes or complications (e.g., *S. aureus* infection, intractable pruritus), will significantly enhance the predictive value of preclinical research (240, 242). Beyond model limitations, current EV-based AD research lacks concrete data on several critical translational parameters. Standardized potency assays that quantitatively link EV preparations to disease-relevant functional outcomes (e.g., suppression of Th2 cytokine release from AD patient cells or restoration of epidermal barrier in AD-like skin equivalents) are rarely reported. Batch−to−batch consistency, a prerequisite for any reproducible biologic product, has not been systematically evaluated across multiple production runs for any EV source in AD models. Biodistribution studies remain qualitative or semiquantitative; it is unclear how many EVs reach the dermis after topical or microneedle application, how long they persist in inflamed versus healthy skin, and whether they are preferentially taken up by specific immune or structural cells. Furthermore, virtually no studies have examined repeat−dosing regimens, despite AD being a chronic relapsing disease requiring long−term management. Long−term safety data — including potential immunogenicity after repeated administration, off−target effects on the skin microbiome, and risks of chronic inflammation or malignant transformation — are absent. Addressing these specific gaps will be essential for advancing EV−based therapies from proof−of−concept toward credible clinical translation for AD.

To evaluate the potential for future clinical application, pharmacokinetics, dosing regimens, and long-term safety are critical issues that will need to be confronted directly in further preclinical and translational studies. Currently, data on the *in vivo* distribution, metabolism, clearance rates, and target organ accumulation of EVs are extremely scarce (243). Routes of administration (topical, subcutaneous, intravenous) and dose selection vary widely across studies, and there is a lack of rational guiding principles for optimized dose exploration (244). Although short-term safety assessments are generally favorable, and xenogeneic EVs have shown tolerability, the potential risks of repeated dosing and long-term application (e.g., immunogenicity, off-target effects) are far from being fully assessed (245). Furthermore, as a “living” biological drug, the manufacturing process (isolation, storage) of EVs directly impacts product attributes and batch-to-batch consistency (246), posing unique challenges for regulatory approval. The U.S. FDA has issued warnings regarding unlicensed exosome products (247), highlighting the urgency of establishing strict Good Manufacturing Practice (GMP) standards and clear regulatory pathways.

Finally, for dermatological treatment, the inefficient transdermal delivery of EVs is a fundamental technological bottleneck. The skin’s stratum corneum is a powerful physical barrier that limits EV penetration, affecting the bioavailability of topical administration. Although systemic administration can circumvent this problem, it leads to non-targeted accumulation of EVs in organs like the liver and spleen. Therefore, developing innovative delivery strategies is a top priority for the future. This includes engineering the EV membrane to enhance skin affinity and penetration capability, as well as leveraging physical permeation enhancement technologies such as soluble microneedles (186), ultrasound, or iontophoresis to assist EVs in crossing the stratum corneum in a non-invasive or minimally invasive manner, achieving efficient and precise local delivery.

In summary, future research on EV therapy for AD should focus on the following key directions: adhering to international guidelines to establish fully standardized processes and strict quality control systems from cell source to EV preparation; utilizing multidisciplinary approaches to deeply dissect the mechanisms of EV action and rationally design next-generation functionally enhanced engineered EVs; developing advanced preclinical models that better simulate the disease spectrum and endotype heterogeneity of human AD; systematically conducting studies on pharmacokinetics, optimal dosing regimens, and long-term toxicology to provide a solid foundation for clinical trial design; and focusing on breakthroughs in delivery technology to develop efficient and safe local administration systems to address the transdermal delivery challenge. Only through interdisciplinary collaboration and systematically addressing these challenges can the immense potential of EVs as next-generation cell-free therapies be fully realized, ultimately bringing new hope to AD patients.
